# Harvesting Practices and Local Ecological Knowledge (LEK) of Bahamian Land Crabs: Bridging Gaps Between Traditional and Scientific Knowledge

**DOI:** 10.3390/ani15202941

**Published:** 2025-10-10

**Authors:** Iain J. McGaw, Michael T. McSweeney, William F. Bigelow, Kaitlyn T. Gaitor, Scott G. Seamone, Owen R. O’Shea, Nicholas D. Higgs, Candice Brittain, Michelle T. Kuenzi

**Affiliations:** 1 Department of Ocean Sciences, Memorial University, 0 Marine Lab Road, St John’s, NL A1C 5S7, Canada; mtmcsweeney@mun.ca (M.T.M.); wfbigelow@mun.ca (W.F.B.); owen@coresciences.org (O.R.O.); 2 Cape Eleuthera Institute, Rock Sound, Eleuthera P.O. Box EL-26029, Bahamas; nickhiggs@islandschool.org (N.D.H.); candicebrittain00@gmail.com (C.B.); 3 Bahamas Agricultural and Marine Science Institute, Queens Highway, San Andros, North Andros, Bahamas; kaitlyngaitortyla@gmail.com (K.T.G.); sseamone@bamsibahamas.edu.bs (S.G.S.); 4 Centre for Ocean Research and Education, 241 Ruth Blvd, Longwood, FL 32750, USA; 5 Department of Political Sciences, University of Nevada, Las Vegas, 4505 Maryland Parkway, Las Vegas, NV 89154, USA; michele.kuenzi@unlv.edu

**Keywords:** *Cardisoma guanhumi*, crabbing, feeding, *Gecarcinus ruricola*, habitat, predators

## Abstract

**Simple Summary:**

This study used surveys to examine the local ecological knowledge and harvesting practices for land crabs across The Bahamas. While crab capture rates and species preferences varied among the islands of Andros, New Providence, and Eleuthera, residents consistently identified similar conservation concerns. Habitat destruction and overharvesting emerged as primary threats to white and black crab populations. Additional challenges included forest fires on Andros and invasive raccoons on Eleuthera. Local crabbers demonstrated a sophisticated understanding of black crab ecology, providing insights that both supported and expanded existing scientific knowledge. Beyond their role as a food source, land crabs held significant cultural and economic value for Bahamian communities, highlighting the importance of sustainable management practices for these species.

**Abstract:**

Three species of land crab occur in The Bahamas; these are an important source of protein and income for Bahamian islanders. The crab harvesters represent an important and largely untapped knowledge source. We conducted surveys on the Bahamian islands of Andros, New Providence, and Eleuthera to document crabbing practices and catalogue this local ecological knowledge (LEK) of land crabs. The survey primarily employed close-ended questions targeting land crab harvesters; we also recorded general feedback from open-ended questions. Crab collection was primarily for self-consumption. Catch rates varied among islands, and were the highest on Andros. There was a preference for white land crabs (*Cardisoma guanhumi*) on Andros, whereas on Eleuthera and New Providence, there was no preference for either white or black crabs (*Gecarcinus ruricola*). The majority of respondents reported a decline in white and black crab numbers, with land development and overharvesting being consistently cited factors. On Andros, forest fires were also reported to account for the loss of crab habitat, whereas on Eleuthera, invasive raccoons were blamed for the population decline. Respondents identified broadleaf forests as critical refuges and food sources for black crabs. Birds were the major predator, confirming findings for other land crab species. Land crabs were not merely a food resource but represented a complex nexus of ecological knowledge, economic systems, cultural traditions, and community practices within Bahamian society. We demonstrated a substantial overlap between traditional and scientific knowledge systems, providing valuable insights into land crab behaviour, habitat use, and ecology that complements formal scientific research.

## 1. Introduction

Brachyuran crustaceans described as land crabs encompass six genera (*Cardisoma*, *Discoplax*, *Gecarcinus*, *Gecarcoidea*, *Johngarthia*, and *Tuerkayana*) and are typically restricted to tropical and subtropical regions [1]. These crabs spend their entire lives on land, returning to the sea only to release their eggs [1]. Breeding migrations may be triggered by seasonal rain, temperature changes, and lunar cycles [2,3]. Once the eggs enter the water, they rapidly hatch into larvae [4,5], which then spend three to four weeks in the ocean during their planktonic development [6,7]. Oceanic larval development ends with metamorphosis into the benthic megalopa stage, which emerges en masse from the sea and migrates back to terrestrial habitats [6,8].

**Figure 1:**
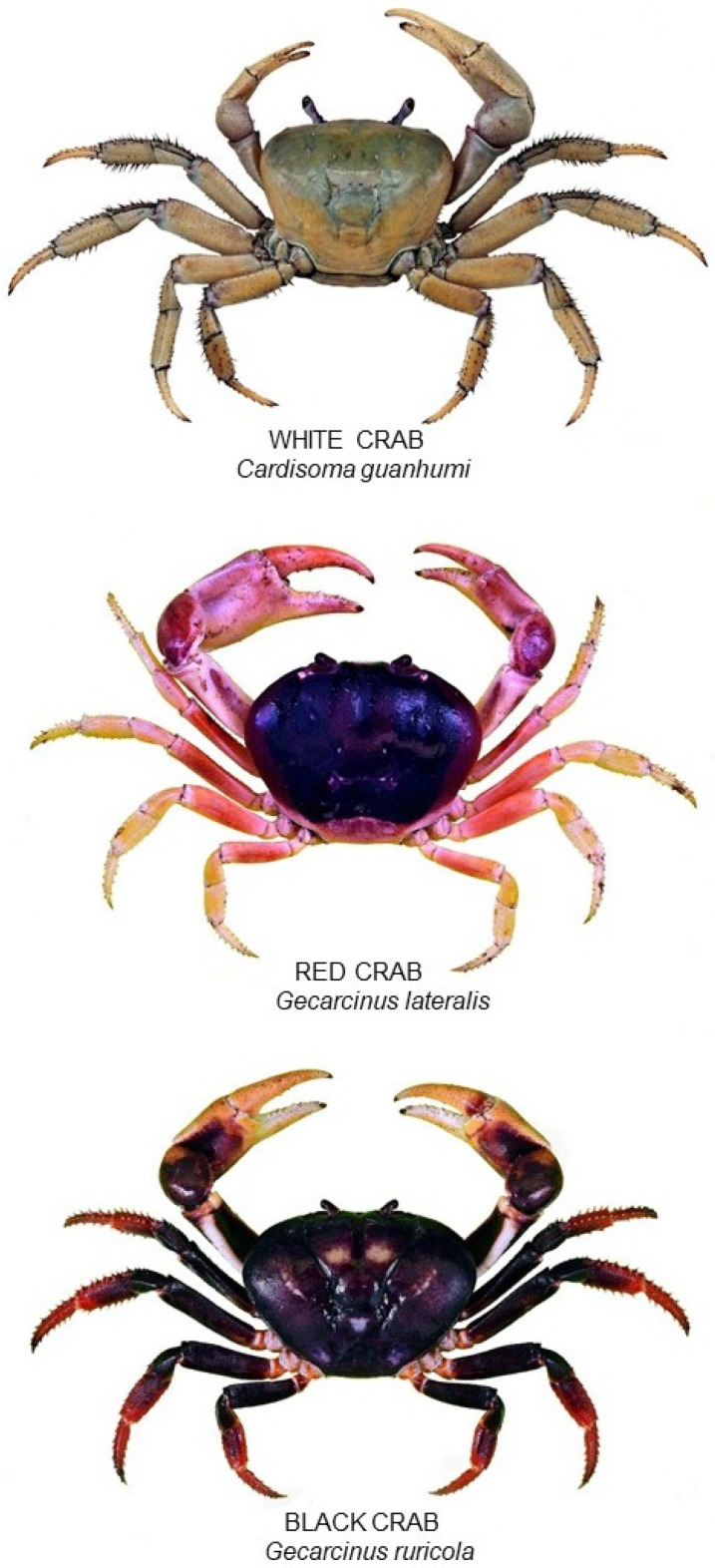
The white land crab (*Cardisoma guanhumi*), red land crab (*Gecarcinus lateralis*), and black land crab (*Gecarcinus ruricola)*. In The Bahamas, the white land crab reaches an approximately 15 cm carapace width (CW), red crabs an 8 cm CW, and black land crabs can grow up to 12 cm CW; images are not to scale (photograph: Iain McGaw).

Within the Bahamian archipelago, three species of land crab are commonly found (Figure 1): the white or blue land crab (*Cardisoma guanhumi*), the red or blackback land crab (*Gecarcinus lateralis*, reclassified *Hartnollius lateralis* in 2025), and the black or purple land crab (*Gecarcinus ruricola*). The geographic range of *C. guanhumi* and *G. lateralis* extends from the northern Gulf of America, through the Caribbean Islands, to Venezuela and Brazil [9,10]. The range of *G. ruricola* is more restricted; it is primarily an island-dwelling species found in the Florida Keys, The Bahamas, and throughout the Greater and Lesser Antilles and Barbados [7,10,11]. *C. guanhumi* is the least terrestrial, typically inhabiting coastal forests near ponds or mangrove swamps, where it digs burrows extending down to the water table [12,13,14]. *G. lateralis* is better adapted for terrestrial life and does not require access to standing water, occupying sand scrublands and coastal forests [2,15]. *G. ruricola* is the most terrestrial of the Caribbean land crabs [6]; they can be found many kilometres inland [11] and at elevations up to 1000 m [15,16,17]. It occupies shaded broadleaf limestone forests, where it burrows in damp soil or shelters beneath rocks and leaf litter [6,7,18]. The more restricted distribution of *G. ruricola* likely contributes to the limited knowledge of its biology and population dynamics compared with *C. guanhumi* and *G. lateralis* [19,20].

In The Bahamas, the artisanal land crab harvest primarily targets *C. guanhumi* and *G. ruricola*, with anecdotal evidence suggesting that the smaller *G. lateralis* is harvested in much lower numbers. All of these species are primarily nocturnal, and as such, most of the harvesting is carried out during the hours of darkness. Unlike other artisanal land crab fisheries in the Caribbean [21], there are few, if any, formal restrictions or regulations on harvest in The Bahamas. Land crabs in other regions (e.g., Indian Ocean coconut crab and Christmas Island blue crab) have become endangered due to overharvesting and habitat loss, and have consequently been placed on protected species lists [22,23]. Similar concerns are emerging in the Caribbean, where climate change, tourism, habitat degradation, and overharvesting may impact the crab populations [24,25,26,27]. In The Bahamas, anecdotal evidence from crab harvesters, along with reports of a 90% population decline over a 40-year period [28,29], prompted the Bahamas National Trust (BNT) to establish the Land Crab Replenishment Reserve on Andros Island to preserve habitat critical for the white land crab (*C. guanhumi*) (https://bnt.bs/explore/andros/crab-replenishment-reserve/ accessed on 16 February 2025). However, less attention has been paid to the black land crab, and generally less is known about its biology and population size [10,19,20]. This is significant because this species comprises the majority of the fisheries on some Bahamian islands, and is not as geographically widespread as the white land crab [20]. The cultural importance of land crabs in The Bahamas was underscored during the COVID-19 lockdowns, when the government permitted crabbing activities to continue.

Beyond their value as a source of food and income, land crabs are also classified as ecosystem engineers [30,31,32]. In The Bahamas, they fulfil a critical role in mixing and disturbing surface soils, similar to the bioturbation of earthworms [33]. They feed primarily on fallen leaves, preventing leaf litter accumulation, which can smother seedlings and impede rainwater infiltration. By digging burrows, crabs aerate the soil and bring leaves underground, introducing nutrients deep into the soil. They also feed selectively on young green shoots, thus influencing the composition of coastal forests [31,34,35]. Climate change projections for the Caribbean suggest not only rising temperatures, but also significant decreases in precipitation [36,37,38]. This is critical because land crabs are far less dehydration-tolerant than their insect cousins and require regular access to freshwater to survive [20,39]. This suggests that future climate change could severely impact populations of land crabs.

While scientists and fisheries officers can monitor crab populations and conduct experiments, the value of local ecological knowledge (LEK) from individuals who interact regularly with these animals is often overlooked [40,41]. Such knowledge is passed down through generations. Historically, incorporating this information into Western scientific research has been challenging [42,43]. Only in the past decade has its importance to the natural sciences been more fully recognised [40,41,44,45,46]. For example, the LEK of Solomon Islanders regarding the Pacific land crab *Tuerkayana hirtipes* has enhanced our understanding of cues driving annual breeding migrations [47]. Similarly, LEK from Brazilian harvesters of the mangrove crab *Ucides cordatus* has provided insights into seasonal abundance changes and effective capture methods [48]. Therefore, the aim of the present study was to engage local Bahamians and develop a two-way knowledge exchange to enhance the understanding of land crab ecology in The Bahamas, with a particular focus on the black land crab *G. ruricola*. Targeted surveys with structured questions were used to document LEK and compare it with the scientific literature to expand knowledge on the abundance, habitat use, and predator–prey interactions of these species. This study focused on crabbing practices, perceptions of population size and sustainability, and local ecological knowledge, especially concerning the black land crab *G. ruricola*.

## 2. Methods

### 2.1. Survey Development

A survey was developed with input from biologists, political scientists, and Bahamian land crab harvesters to collect and collate the local ecological knowledge (LEK) of Bahamian land crabs. The surveys were conducted one-on-one by Interdisciplinary Committee on Ethics in Human Research (ICEHR; Memorial University of Newfoundland)-certified individuals in various settlements, primarily across the Bahamian islands of New Providence, Eleuthera, and Andros, with a few collected from Great Exuma, Grand Bahama, Long Island, Abaco, and Cat Island. Surveys were carried out in 2022 (Eleuthera) and in 2024 (Andros, New Providence, Eleuthera, other family islands). The survey targeted people who were active crabbers, had gone crabbing before, or were involved in the land crab industry (e.g., processors or restaurant owners). We advertised the survey via social media and word of mouth and arranged a time and place for participants to meet. No compensation was given for completing a survey. Surveys mainly consisted of closed-ended structured questions, with a few open-ended questions allowing the respondents to provide detailed feedback (Supplementary Data S1). Additional details or comments were sometimes volunteered, even for closed-ended questions. A total of 112 surveys were collected. Some participants did not respond to every question. The number of responses was reported as a percentage of total responses received for each question, with exact numbers given in parentheses. All data were presented as the means ± standard deviation. Statistical significance was accepted at the *p* < 0.05 level. All surveys were carried out under the guidelines of the ICEHR permit 20250055-MI (15 May 2024) and the Government of The Bahamas, Department of Environmental Protection and Planning (permits, BS2022-573928 and BS2024-929848).

### 2.2. Survey Questions

For the present study, we focused on questions related to crabbing practices in The Bahamas, the cultural value of land crabs, and local ecological knowledge. Other topics in the survey, such as attitudes, societal trust, and potential regulations in relation to demographics, were analysed separately (Kuenzi et al., In prep). Some questions (13–17, 20, 23–24, 36, and 39) were analysed as a function of the respondent’s island of residence. Eleuthera is an island characterised by inland broadleaf limestone (blackland) coppice and coastal sandland coppice and scrub [49], and is known for its abundance of black land crabs [20]. Andros consists of a mix of pine forests, mangroves, and coastal ponds [49], and also contains a 4000-acre white land crab replenishment reserve in central Andros, established to protect and enhance white land crab recruitment [19]. New Providence, which includes the capital Nassau, is the most developed island and is primarily composed of pine forests and dry coastal scrub [49]. While land crabs appear to be less abundant on this island, more crabs are shipped to New Providence for processing [50]. We also obtained nine surveys from other Bahamian islands. As these did not constitute sufficient sample sizes for robust statistical analysis, they were assigned to one of the three primary island groups based on habitat similarity and/or level of human development. Great Exuma (*n* = 4), Cat Island (*n* = 1), and Long Island (*n* = 1) were grouped with Eleuthera. Freeport, Grand Bahama (*n* = 2) was grouped with New Providence, and Abaco (*n* = 1) with Andros. For questions 8, 10, 19, 21, 25, and 40, which addressed more general aspects of land crabs, or questions that pertained specifically to black land crabs (*G. ruricola*) [32,33,34,35,37,38], data from all island groups were combined.

### 2.3. Data Processing

There was a large variation in the reported daily catch rates; therefore to try and standardize crab catch data, an estimated catch per unit effort (# crabs/person/hour) was calculated by dividing the number of crabs caught (Q13) by the hours spent crabbing (Q12). When a range was provided, the mean of the two values was used. This value was further divided by the number of people crabbing (Q10) using the following values: on their own = 1; with a partner = 2; with friends = 3; with family = 4. These group sizes were informed by observations indicating that groups of “friends” typically included 2–4 people, while “family” groups tended to be slightly larger (Bigelow, Seamone, pers. obs.). The Data were not normally distributed and so a Kruskal–Wallis test (one-way ANOVA on ranks) was used to compare groups. If a significant result (*p* < 0.05) was obtained, Dunn’s pairwise comparison tests were used to determine where specific differences occurred. For questions (15–17, 20, 24–25, 36, 39) that involved comparisons across islands and for those with varying degrees of responses (19, 21, 25), Chi-square tests were used. If significance was found, Bonferroni post hoc pairwise comparisons were performed to identify where differences occurred. Data for questions 33–35 and 37 were described in terms of proportions. All statistical analyses were carried out using SigmaStat version 4.

Open-ended responses as well as additional feedback provided for closed-ended questions, were analysed thematically [51,52]. Semantic thematic analysis was selected because the participant responses were generally short and direct, consisting of single words or brief sentences. This approach was considered appropriate, as it assumes no underlying or hidden meanings behind the responses [53]. We used the AI program Claude to conduct an initial semantic thematic analysis and assign numerical frequencies to reported themes. The themes were then manually checked and validated; any anomalies were reanalysed with additional instructions regarding the grouping of responses. This process generated a considerable volume of output. In the Results section, we summarise the most frequently reported or noteworthy responses and include illustrative quotes. Full thematic analyses for individual survey questions are provided in Supplementary Data S1.

## 3. Results

### 3.1. Crabbing Practices

*Q11: Who do you normally go crabbing with?* Ninety percent (94/104) of respondents went crabbing with family or friends. Five percent (5/104) of respondents went with a crabbing partner and 5% of respondents (5/104) crabbed alone. It was suggested that crabbing in groups was more successful because you could “surround them and herd them.”

*Q12: How many hours do you typically go crabbing on a given night?* The time spent crabbing could be as brief as 1 h and up to 18 h (day and night), if going back deep into the forest. On average, people spent 4.2 ± 2.7 h (SD) crabbing per night (*n* = 104).

*Q8: In which season do/did you go crabbing.* We received 91 responses, 86.8% (79/91) responded as summer only, 13.2% (12/91) crabbed in both summer and winter, and no one went crabbing in the winter only.

*Q10: Where and how do you catch land crabs?* The majority of people went crabbing in multiple places: 73.1% (76/103) crabbed on the side of the road, 61.5% (64/103) deep in the bush, and 16.4% went digging for crabs (17/103); four “other” responses (3.9%) listed “ponds, marshes, and swamps” as places to collect crabs but these were for white crabs only. Those that discussed digging for crabs said it was carried out off-season or in the winter when crabs were buried and not feeding.

*Q13: On a good night how many do you catch?* All respondents simply reported the total number of crabs they caught during a good night of crabbing, but did not break down the actual numbers by species or sex. When comparing the catch across islands, there was a significant difference among islands, and even a large variance within individual islands (Kruskal–Wallis, (df = 2), H = 22.60, *p* < 0.001). Andros (*n* = 32) had a mean catch of 215 ± 183 (SD), which was significantly higher (Dunn’s test, *p* < 0.01) than both New Providence (*n* = 21) with a mean catch of 70 ± 96 crabs and Eleuthera (*n* = 52) with 69 ± 101 crabs; the catches on these two islands were similar to one another (Dunn’s test *p* > 0.05). The estimated CPUE followed a similar pattern (Kruskal–Wallis, (df = 2), H = 17.22, *p* < 0.001), with Andros having the highest estimated CPUE of 15.2 ± 13.3 crabs/h/person; this was higher (Dunn’s test *p* < 0.01) than both the 5.0 ± 6.7 for New Providence and 6.6 ± 7.9 for Eleuthera, which were similar to one another (Dunn’s test *p* > 0.05).

*Q14: What kind of land crabs do you catch?* The respondents did not break down the actual numbers of each species of crab. Overall, across the islands, 42% of crab harvesters collected both white (*C. guanhumi*) and black crabs (*G. ruricola*), 28% caught white only, 16% caught black only, and 13% collected white, black, and red land crabs (*G. lateralis*), with 1% of crabbers targeting white and red crabs. No crabbers reported taking just black and red land crabs. This equates to 84% of people surveyed harvesting white crabs, 71% taking black crabs, and 14% collecting red crabs.

**Figure 2:**
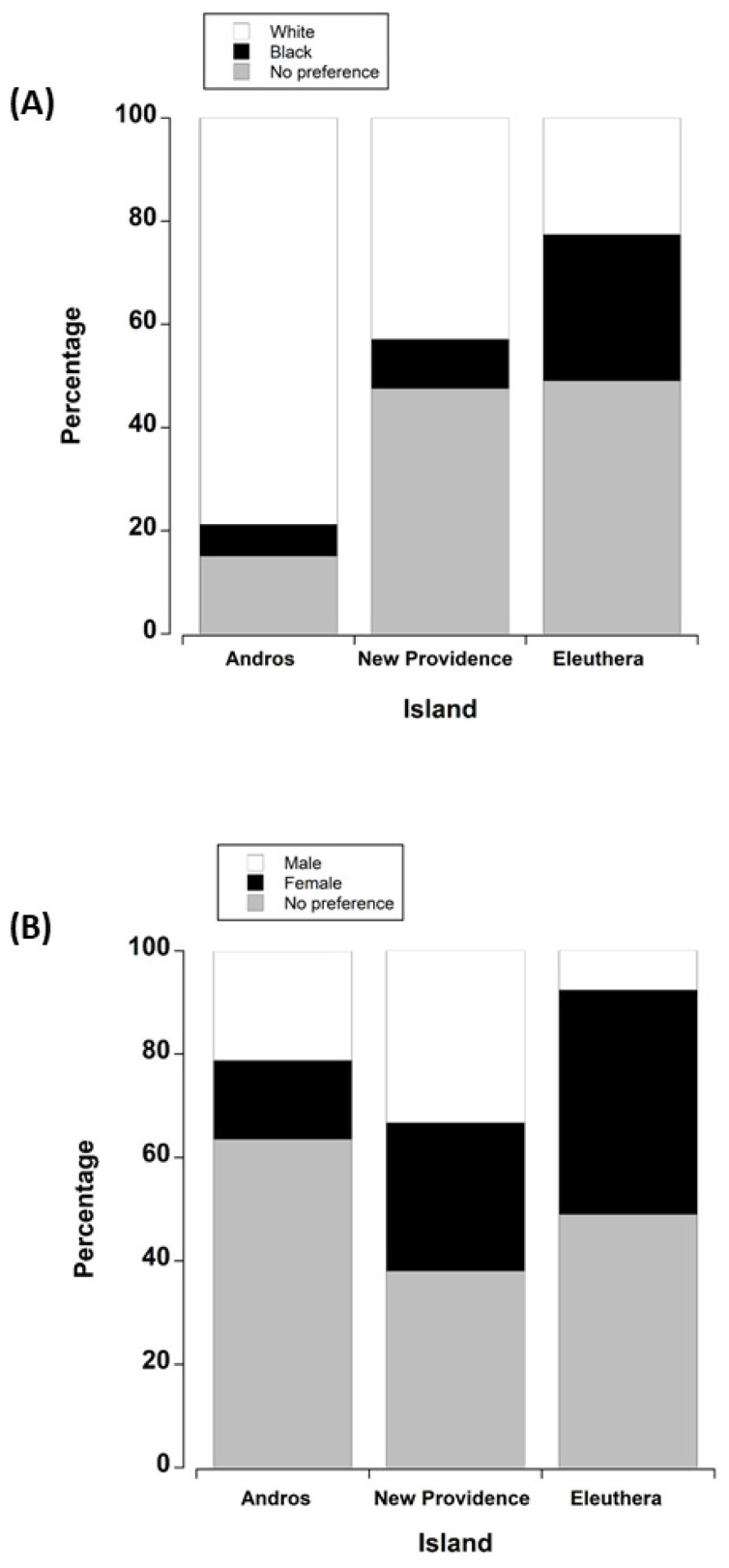
Percentage of people across the islands of Andros, New Providence, and Eleuthera who expressed a preference (or otherwise) of catching (**A**) white or black land crabs and (**B**) male or female crabs. Chi-square analysis was carried out on the actual number of respondents, but for visualisation purposes, these were converted to percentages for each island.

*Q15: Do you prefer to catch white or black crabs, or have no preference?* We received 107 responses and there was a difference across the islands (ꭓ^2^ = 20.3 (df = 4), *p* < 0.001). The differences occurred between Andros and Eleuthera only (Bonferroni, *p* < 0.001). Crabbers on Andros appeared to have a strong preference for white crabs compared with crabbers on Eleuthera, the majority of whom did not have a preference for either species of crab (Figure 2A). The responses of Eleuthera and New Providence and Andros and New Providence were similar to one another (Bonferroni, *p* > 0.05).

When people expressed a preference, thematic analysis showed regional differences with the highest frequency of responses based on taste (Supplementary Data S1). Twenty-nine percent of people stated that white crabs had a “better taste” (without specific flavour descriptors) and had “more fat”, whereas 37% of respondents said black crabs were “sweeter tasting.” The larger size of white crabs, and thus more meat, was given by 20% of people as the second most important reason for their preference. White crabs were most often eaten on their own, and black crabs were usually made into recipes with something else like rice. White crabs were more consistently described as being more profitable when sold and more abundant overall, although black crabs were more common in specific areas. Finally, there were differences in catchability, despite being faster, white crabs were reported as easier to catch and less feisty, while black crabs were described as “hostile”, although the smaller claws made them easier to handle. Red crabs stood apart as primarily utilitarian rather than culinary, with their main value being as fishing bait. These were often referred to as soldier, diddler, joombie, or gallen crabs.

*Q16: Do you prefer to catch male or female crabs, or have no preference?*

We also received 107 responses to this question. There was a difference across islands (ꭓ^2^ = 13.42 (df = 4), *p* = 0.009). A post hoc Bonferroni test showed that these differences occurred between Andros and Eleuthera only. On Andros Island, there was not a strong preference for either sex, whereas on Eleuthera, crabbers appeared to prefer to catch female crabs (Figure 2B). No significant differences between New Providence and either Eleuthera or Andros were detected (Bonferroni test, *p* > 0.05). Overall, more people preferred female crabs. Thematic analysis showed that the main reason for this preference (75%) for female crabs was the eggs, which were used in various recipes. Fifty-eight percent of respondents said the higher fat content of females was also very important; both eggs and fat imparted a sweeter taste, and they had more culinary uses. On the other hand, the absence of eggs and the dislike of eggs was the key reason (50%) for preferring male crabs. Males were also preferred (36%) because they were larger, with larger claws and therefore had a higher meat yield. Although people often expressed a preference for catching black or white crabs, or males or females, they also stated that they usually just took what they found.

**Figure 3:**
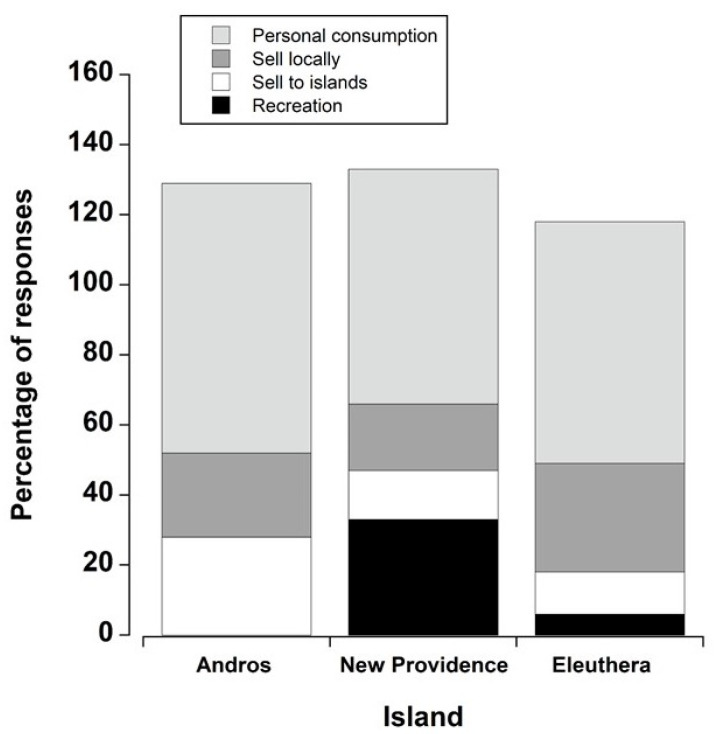
Potential use for land crabs (once captured) as a function of the island of capture. Crabs were eaten by the harvesters, sold within the local community, or sold to other islands. Recreational uses included catch and release for entertainment, or for contests such as the biggest crab, or in crab races. Chi-square analyses were carried out on the raw count data. For visualisation purposes, the data are shown as the percentage of total responses for each island. Because individuals quoted numerous uses, the percentages do not total 100%.

*Q17: What do you do with the crabs you catch?* We received 101 responses, with most people citing multiple uses for crabs. We combined “personal consumption” and “share with family and friends” because when individual crabbers ate them, it was invariably with their family and friends. There were differences among the islands (ꭓ^2^ = 17.39 (df = 6), *p* = 0.008). Across islands, most of the crabs were collected for personal consumption. There were differences between Andros and New Providence (Bonferroni, *p* < 0.05), but uses were similar when comparing Eleuthera with both New Providence and Andros (Bonferroni, *p* > 0.05). Crabbers on Andros either consumed the crabs or sold them (presumably for consumption), whereas a relatively high proportion (30%) of crabbers on New Providence used crabs recreationally (Figure 3): A number of people practiced catch and release, stating that it was good exercise to get out in the bush for crabs; others used them for biggest crab contests or crab races. Crabbers also detailed husbandry practices; 15% of people who offered additional feedback said crabs had to be kept in pens for two weeks to two months and fed mangoes, coconuts, or “dilly” leaves prior to consumption or selling. This time allowed for flushing or cleaning crabs (stated by 24% of respondents) and was used not only to remove waste, but also to impart a sweeter taste. It was also important to make sure that crabs were kept out of the heat, had a water supply, and were not fed cooked food; all of these factors were reported to increase longevity in captivity. It was notable that 24% of respondents said that land crabs (white crabs in particular) were an important dish for Father’s Day celebrations.

**Figure 4:**
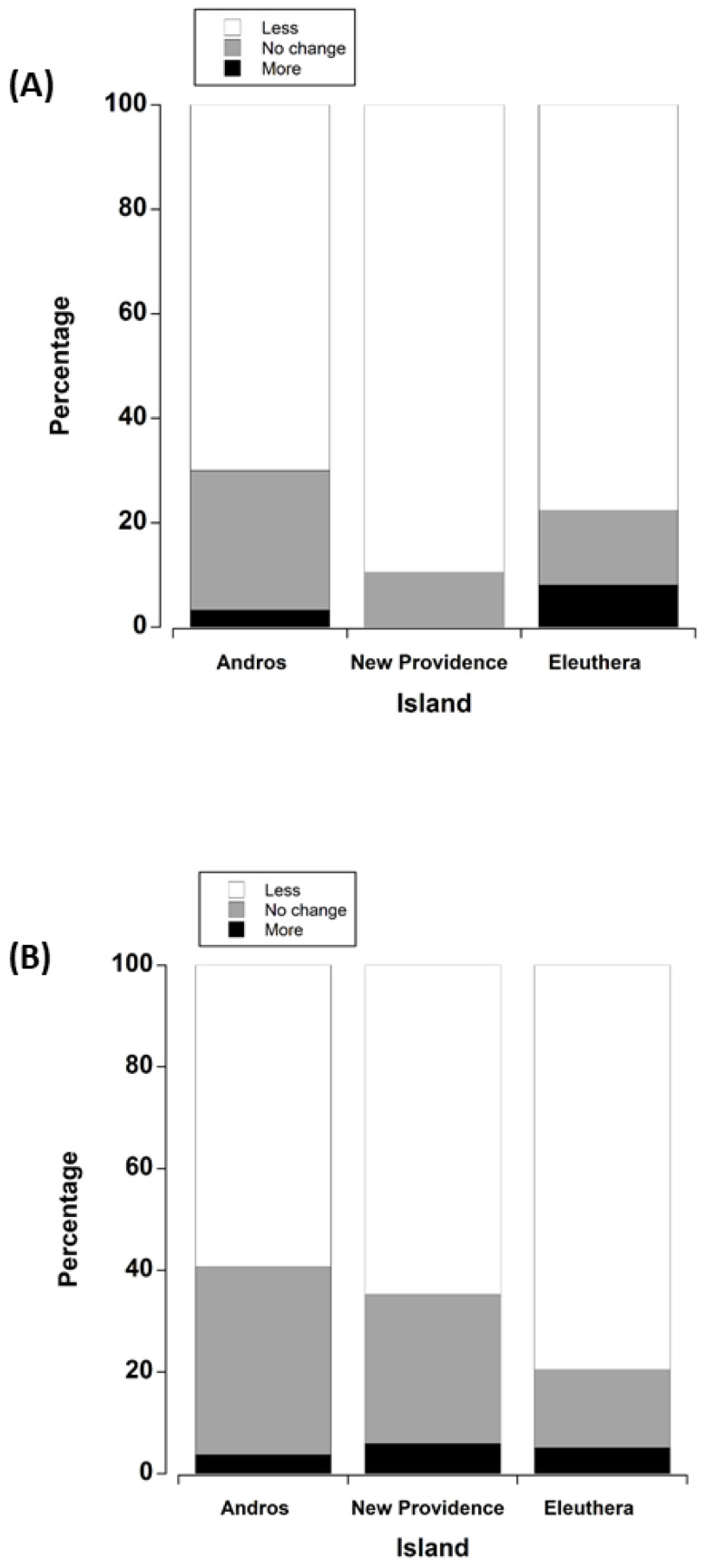
Percentage of people across the islands of Andros, New Providence, and Eleuthera who reported less, no change, or an increase in the abundance of (**A**) white land crabs and (**B**) black land crabs. Chi-square analysis was carried out on the actual number of respondents, but for visualisation purposes, these were converted to percentages for each island.

*Q23: Do you think there are more or less white crabs on this island than there were five to ten years ago, or has there been no change?* We received 103 responses across islands; five “do not know” responses were not used in the analysis. For white crabs, there was no significant difference in responses among islands (ꭓ^2^ = 4.90, (df = 4), *p* = 0.298). However, a one sample z test on the proportions (z = 10.49, *p* < 0.001) showed that the majority of people on each island reported that there were less crabs, and only a few people in each case reported an increase in the number of white land crabs (Figure 4A). Although there were no differences among islands, the ideas behind the decline on each island differed (thematic analysis). On Andros, harvest practices (57%) were the dominant concern, specifically the number of people targeting spawning females with eggs (43%). Forest fires (38%) were also seen as a major threat unique to Andros. On New Providence, development and urbanisation was the overwhelmingly dominant (75%) reason given for the decline in white crab abundance. There was minimal mention of harvest practices (5%) having any effect on New Providence. On Eleuthera, invasive raccoons (32%) and human harvesting practices (32%) were of equal and high concern. The unique focus on raccoon predation was not emphasised on other islands. Urban development (29%) was also cited as having a dominant effect on the population of white land crabs on Eleuthera. Some people on Eleuthera perceived the decline in white crabs was caused by Haitian immigrants (11%) harvesting crabs, and harvest by illegal immigrants was voiced (to a lower degree) across the other islands.

*Q24: Do you think there are more or less black crabs on this island than there were five to ten years ago, or has there been no change?* We obtained 93 responses, ten of these were “do not know” and these were not used in the analysis. Similar to white land crabs, there were no significant differences in changes in black crab abundance among islands (ꭓ^2^ = 4.72 (df = 4), *p* = 0.375) (Figure 4B). However, the overall response was that there were significantly less black land crabs on each island than had occurred previously (z test, z = 7.26, *p* < 0.001), although the percentage of people who reported less black crabs was a little lower compared with reports for white crabs. Thematic analysis showed somewhat similar perceived reasons for the collapse of black land crab populations. On Andros, habitat destruction by forest fires (33%) and overharvest of reproducing females with eggs (33%) were cited as key reasons for decline. However, an increase in predation (17%) on black crabs was also cause for concern. On New Providence, urbanisation and land development and associated deforestation (50%) were the main reasons for the decline; only 8% cited overharvesting, as there was very limited commercial activity. On Eleuthera, the main reason for black crab decline was suggested as land development, namely clearing forests. As with white crabs, raccoons (41%) were also thought to prey on crabs, reducing their numbers. Twenty-nine percent said that an increased demand for black crabs and more harvesting and digging of crabs was reducing their numbers. Unlike white crabs, approximately 25% of verbal feedback expressed hope for black crab populations, suggesting that they were still seeing plenty of baby crabs and one just needed to go further back in the bush to find black crabs.

**Figure 5:**
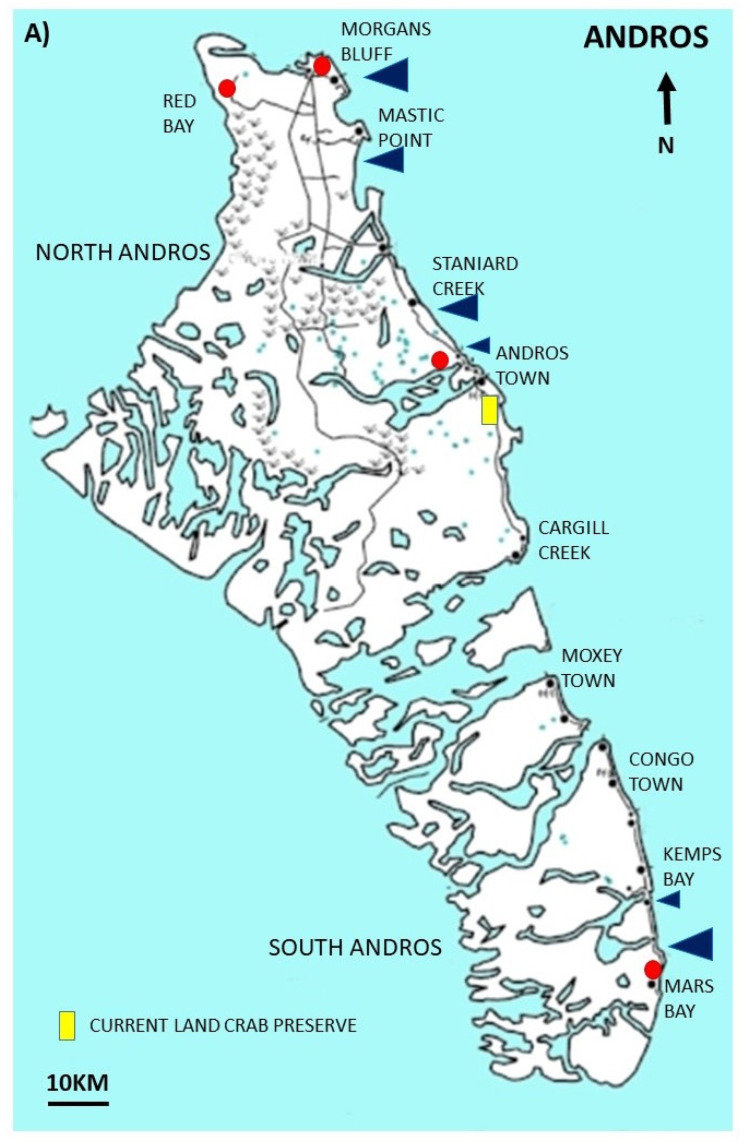

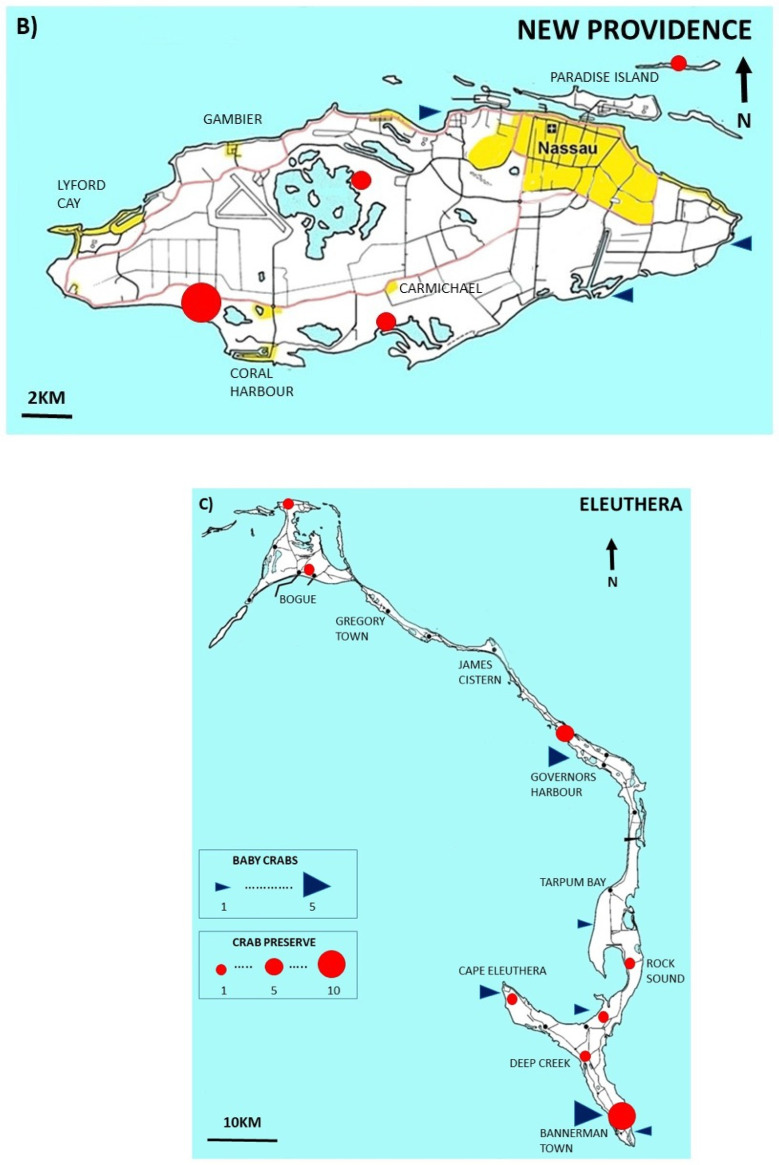
Annotated maps of the Bahamian islands of (**A**) Andros, (**B**) New Providence, and (**C**) Eleuthera. The blue triangles mark the reported locations of sightings of baby crabs returning from the ocean (Question 36), the larger the triangle, the more people reporting sightings at that location. The red circles are locations where people supported the introduction of a black land crab preserve, where crabs could not be harvested (Question 39). Again, the larger the circle, the more individuals suggested the locale.

*Q36: Have you ever seen baby land crabs returning from the sea?* We received 95 responses in total; three “do not know” responses were not used in the analysis. There was a significant difference among islands (ꭓ^2^ = 12.98 (df = 2), *p* = 0.002): 50% (14/28) of people had seen them on Andros, 48.9% (23/47) of people had observed them on Eleuthera, and significantly less people—10% (2/20) said that they had seen baby crabs on New Providence (Bonferroni test *p* < 0.05). The locations of where the crabs were observed are marked with blue triangles (Figure 5). Not all respondents provided a timeline, but approximately 60% (18/31) of people had seen them in the last one to two years. Six respondents reported seeing crabs 3–6 years prior to being interviewed, and four people reported that it had been a long time since they had seen baby crabs returning from the ocean (10–40 years) with the statement “It was raining crabs 40 years ago here on Eleuthera”.

*Q20: How important are land crabs to the economy of your island?* There were different responses across the islands (ꭓ^2^ = 24.24 (df = 8), *p* < 0.001) (Table 1). On Andros, all responses skewed towards a high importance, and no one said they were not important or slightly important. On New Providence, the opposite response occurred with <10% of people reporting them as very important, and no one said they were extremely important, whereas on Eleuthera, there was a more even spread of responses (Table 1). The responses for Andros were different to those from both New Providence and Eleuthera (Bonferroni test, *p* < 0.001), while the responses for Eleuthera and New Providence were not significantly different from one another (Bonferroni test, *p* > 0.05).

### 3.2. General Perceptions

*Q19: How important are land crabs to Bahamian culture?* Land crabs were obviously a cultural commodity (Table 2), and only 0.9% of respondents said that they were not at all important, but nearly 90% of respondents said that they were very or extremely important to Bahamian culture (Table 2). In addition to the importance of eating crabs for celebrations like Father’s Day, or iconic dishes like crab and rice, there was also a social aspect of getting together to crab. It was mentioned by a number of people that it was less common now for the youth to go crabbing.

*Q21: How important are land crabs to the Bahamian economy?* When asked about the economic significance, there was a broader distribution of answers, however, there was still a significant non-random pattern, and the majority of feedback (32.7%) still listed them as extremely important to the economy of The Bahamas. Five of the respondents who listed “not at all”, or “slightly important” did say that it depended on the specific island (Table 2).

*Q25: Potential threats to land crab populations*. Respondents were offered a choice of graded responses, if they responded as “don’t know”, these were not used in the statistical analyses (Table 3). All responses except for the question on whether invasive species would impact land crabs (25 D) exhibited a non-random pattern (ꭓ^2^, *p* > 0.05). Seventeen and a half percent of respondents did not think that climate change would affect the land crab population, whereas 50% of respondents did think that it would have an effect on the population sizes of land crabs. For overharvesting, the pattern was somewhat similar, with 22.5% disagreeing that overharvesting would have any effect, while 48% of respondents thought that it would affect the crab population size in the future. The clearest responses were for land development: only 8.8% of people did not think that land development would affect land crabs, whereas 87.6% agreed that it would harm land crabs. When asked about the potential affect invasive species would have on land crabs, the responses were more varied, with no clear consensus on what the future effects might be. Finally, when asked if there would be fewer land crabs in the future, 23.8% did not think that there would be fewer crabs, but the majority (56.3%) were concerned about a diminished population of land crabs (Table 3).

### 3.3. Black Land Crab Biology

*Q32: Where do you find black crabs in forest/bush?* This open-ended question received 96 responses; most people did not go into any detail, only giving a few words describing general habitats. A thematic analysis showed four main clusters of responses. The majority of responses suggested that concealment/cover (53%), whether under rocks, fallen logs, or trees, was the most important. Underground structures, such as holes, burrows, and limestone sinkholes (37%), were also commonly cited as important habitats for black land crabs. A similar high percentage (36%) discussed that crabs were found under or around specific trees or crops and that the procurement of food (9%) might be important. In a more detailed description of the forests, 29% of people said that “thick/dense forest” and “deep in the bush” were where black crabs lived. Finally, within these forest environments, moisture was deemed important, as 13% listed swamps, marshy areas and/or ponds.

*Q38: Why do you think the forest/bush is important for black crabs?* One hundred out of one hundred and one people said that the forest is important for land crabs, and all gave reasons. Only one person answered no, and did not give a reason for this answer. The primary response (62%) given did not detail any reason, simply stating that is “where they live”, or “it is their home or habitat”. The second most important resource (28%) was for protection; the forest provided crabs somewhere to hide from predators and humans. Nineteen percent of people suggested that the bush was important for feeding on fruits, berries, and plants. Finally, the forest provided shade and coolness from the environment (presumably hot and dry conditions), where the crabs could burrow into the soil (11%).

*Q33: During which season do you see most black crabs running?* Ninety-three point three percent (97/104) of people said that they saw most black crabs running during the summer, 2.9% (3/104) reported both summer and winter, 2/104 (1.9%) said black land crabs were active during the winter, and 1.9% (2/104) reported that they “didn’t know” when black crabs were running. A number of people gave specific timelines suggesting that you could start seeing black crabs as early as April to May, but numbers noticeably increased from June onwards.

*Q34: Do you see most black land crabs running when there is a full moon or a new moon?* The responses concerning the phase of the moon were very diverse: 24/86 (27.9%) of people said that black crabs were most active during the full moon; 21/86 (24.4%) said the new moon, and 41/86 (47.7%) reported not knowing the moon phase when black crabs were running. For both of these questions, some specific respondents stated that heavy rains had to coincide with total darkness, and the best crabbing was on the darkest nights about a day after heavy rains. It was also stated that after heavy rains, black crabs could be seen out and active on the surface during the daylight hours.

**Figure 6:**
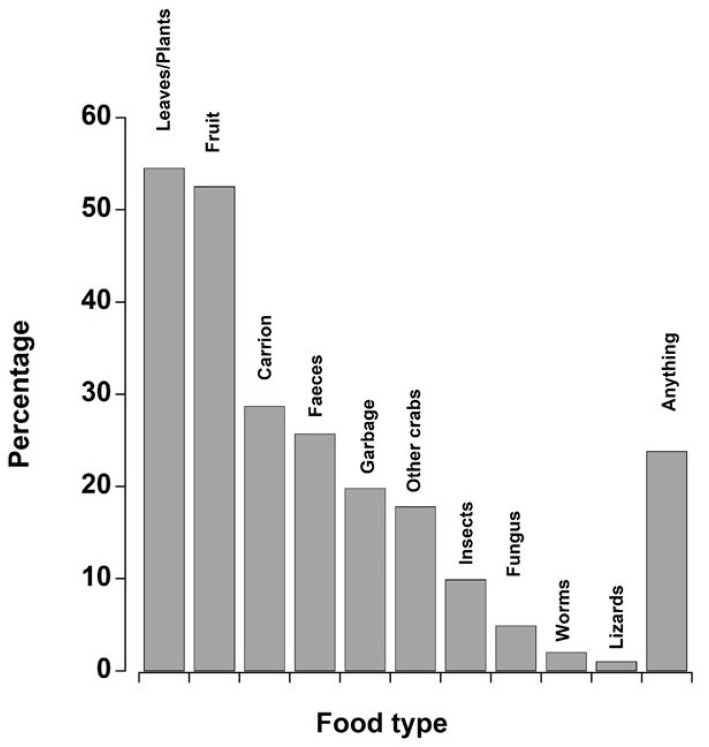
Graph showing the food items that the respondents suggested black land crabs feed upon across all islands surveyed. For visualisation purposes, the data are shown as the percentage of total responses, and because individuals usually named more than one food item, the percentages did not total 100%. A comparatively large proportion of answers were “anything”, and so this was assigned its own separate category.

*Q35: What do you think black crabs eat?* We received 101 responses, with most people listing multiple food types. A significant proportion of people said “anything”, so this was added as a category. Over 50% of people responded that black land crabs ate leaves and fruit, and between 20 and 30% responded as eating carrion, faeces, garbage, or anything. Eighteen percent of people suggested that they were cannibalistic, eating other (smaller) crabs. Less than 10% of respondents suggested that crabs ate other forest animals such as insects, worms, and lizards (Figure 6).

**Figure 7:**
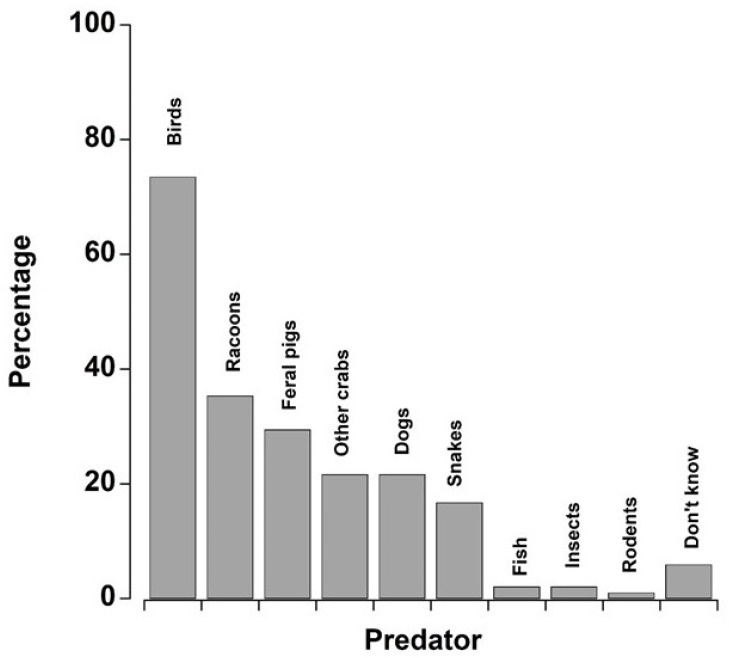
Graph showing the potential predators that respondents suggested eat black land crabs (data for all islands combined). For visualisation purposes, the data are shown as the percentage of total responses, and because individuals usually named more than one predator, the percentages did not total 100%. A comparatively large proportion of people did not know what might eat black crabs, and so this was shown on the graph.

*Q37: Other than people, what eats black crabs?* We obtained 96 responses, with people usually listing multiple animals that would prey on black land crabs. A clear majority of people (73.5%) listed birds as the main predators of black land crabs. Next was invasive species: feral pigs and raccoons were listed as important predators. Roughly equal percentages (17–22%) listed snakes, other crabs, and dogs. A low percentage of responses (<2%) listed insects, fish, and rodents as predators of black crabs. It was notable that 6% of respondents did not know what might eat black land crabs (Figure 7).

*Q39: Do you think there should be an area of forest on the island that should be a black crab preserve where they cannot be harvested?* There was no significant difference as to whether people thought there should or should not be a black land crab preserve on their island (ꭓ^2^= 4.90 (df = 2), *p* = 0.087). Although slightly more people replied with a positive response (56% or 51/91), this was not significantly different from the number of people who did not want a black land crab preserve (z test, z = 1.15, *p* = 0.245). Twelve individuals did not know whether there should be a preserve on their island, and this was not factored into the statistical analysis. For people who agreed that there should be a black land crab preserve, the possible general locales are indicated on each island map with a red circle (Figure 5).

### 3.4. Other Feedback

*Q40: Are there any other things about land crabs that you would like to share with us*? For this question, we received similar information to that volunteered for some of the other questions. However, there were still unique insights, which the thematic analysis defined into three broad categories.

(1) Ecological knowledge, where 20% of responses discussed the crab habitat. Crabs do not like dry areas, vibration, sound, or heat, and the backs of crabs are always wet with moisture. Crabs march twice a year and lay their eggs on a high tide. They moult in their burrows and should be protected from harvest during this time. Baby crabs live in the burrow with adults, and the juveniles hang out near burrows. Sixteen percent of people noted interactions between white and black crabs with statements like “the white crabs hate black crabs”. White crabs are dominant and attack and eat black crabs so much that they cannot be kept in the same pen.
(2) Cultural and culinary knowledge: We already documented in previous questions that land crabs were an important food resource across The Bahamas, but this additional feedback suggested that they tasted different from island-to-island, and that different cultures had different recipes. Crabs are culturally important; most crabbers are now older, but it was important for the youth to learn crabbing. On Eleuthera, it was stated that “Bannerman town is the crab Mecca”, “where houses were built off crabbing”. Andros was also mentioned as being an important island for crabbing and “crab fest”.
(3) Harvesting and management practices: Fourteen percent of people discussed methods of catching crabs, stating that they could be dug up, but that it was detrimental if they were dug up during the winter. A number of responses suggested that once a house was built with a farm or garden, the crabs would come in to eat the fruit. It was suggested that in these instances, the crabs could become an agricultural pest. In addition, Bahamians indicated an underlying tension with Haitian migrants who also harvested land crabs, raising concerns over access and control.

## 4. Discussion

This study collated hitherto unrecorded information regarding the crabbing practices and ecology of land crabs in The Bahamas. Overall, the feedback revealed that land crabs were not merely a food resource, but represented a complex nexus of ecological knowledge, economic systems, cultural traditions, and community practices within Bahamian society [29,54].

### 4.1. Crabbing Practices

Responses to multiple questions showed that land crabs were evidently very important to Bahamian culture, which was not unexpected, as the LEK of harvested species is usually far more extensive than that of non-harvested ones [45]. Although the primary reason for harvesting crabs was for self-consumption, crabbing also had important connotations for social bonding, with the majority of people crabbing with family and friends [55,56]. Many of the references were to food, with the catch being eaten by the crabbers and/or the local community not just as a source of protein, but also as culturally significant dishes. The overwhelming association with Father’s Day suggests a deeply embedded cultural tradition [54], linking crab consumption specifically to this holiday and representing significant cultural patrimony. Two-thirds of people surveyed on Andros said crabbing was culturally important, and although some people on Andros made a moderate living selling white crabs (*C. guanhumi*), the marketing of black crabs (*G. ruricola*) was of limited economic importance on the Bahamian islands we surveyed (Kuenzi et al., In prep). This is in contrast to other islands in the southwestern Caribbean (Old Providence and Santa Catalina), where the harvest of black crabs is an important source of income, with approximately 1.2 million crabs being captured each year [25]. A recent paper confirms the findings that white crabs are more highly exploited in the northern and eastern Caribbean, but black crabs are more important in the western Caribbean [25].

Red crabs (*G. lateralis*) stood apart as primarily utilitarian rather than culinary, with their main value being as fishing bait. Specialised local names for red crabs indicated their cultural recognition despite limited food value. Fishermen often have specific language and descriptions for their catch [57], and The Bahamas are no exception. For example, the local name for red crabs (joombies/jumbees) has spiritual connotations, as “jumbee” typically refers to spirits or ghosts in Caribbean folklore, possibly reflecting these crabs’ quick, erratic movements [58]. Red crabs were also referred to as the gallin/gaulin crab because they are eaten by gaulin birds (night and grey herons; [59]). White or “pond” crabs are so named because they construct deep burrows with standing water at their base and are commonly found close to mangrove swamps or wetland areas [13,14].

When asked about their preference for catching white or black crabs, crabbers on the island of Andros stood apart in expressing a strong preference for white crabs, whereas on the other islands, approximately half of the people surveyed expressed no preference, simply stating that they would take what they could catch. Andros is the largest island in The Bahamas, and white crabs are the dominant species because of the expansive mangrove swamps and wetland habitats [13,60]. According to The Bahamas Ministry of Tourism (2010), “Crabbing has long been a fueller of Andros’ economic engine and some of the nation’s best doctors, lawyers, teachers, and politicians can trace their roots to the industry”. Andros is also the site of the white crab replenishment reserve and the annual crab festival, which takes place every June [29]. Where people did express a preference for either white or black crabs, taste was the primary reason, with white crabs tasting “better” or black crabs tasting “sweeter”. These seemingly contradictory responses are subjective and simply individual preferences to unique taste and odour profiles [61]. One person suggested that crabs taste different on different islands, a fact also reported for islands in The Colombian Archipelago of San Andres [26]. Many crabbers reported holding crabs for long periods (weeks to months) and feeding coconut or mangoes to “impart a sweeter taste”. This practice is widely used for Chinese mitten crabs (*Eriocheir sinensis*), mud crabs (*Scylla* species), and even some land crab species, whereby the fat content and amino acid profiles and thus flavour are changed according to consumer preference [62,63,64,65]. There was also reference to white crabs being larger (more meat) and more marketable than black crabs. Certainly, between 500,000 and 700,000 white crabs are shipped from Andros to New Providence and the Family Islands on an annual basis [19,29].

When asked about preference for male or female crabs, the majority of respondents on each island expressed no preference. Where preference was expressed for females, it was primarily to do with taste related to the presence of eggs and a higher fat content. This made females more flavourful and thus more versatile for cooking. On the other hand, the lack of eggs and fat (taste) was also the primary reason for the preference for males. A number of respondents suggested avoiding crabs when they were spawning. It was unclear whether this was related to conservation—with crabbers avoiding females thereby allowing them to release their eggs—or specific changes in palatability during the breeding migration. Crabbers on the Colombian island of Old Providence have reported that crabs have a poor taste after spawning [25], and on Andros, social media is being used to advertise when and where the breeding migration is occurring [19]. Both of these suggest more pressure to capture crabs before/during the breeding migration, rather than conservation concerns. For male crabs, the second most common reason for their preference was their larger body and higher meat yield. On Andros Island, where white crabs make up the bulk of the harvested species, there was a slight skew towards male preference, and males were harvested at a ratio of 2:1 compared with females [19,29]. White crabs show a clear sexual dimorphism: males are not only larger, but they also possess a larger heterochelous claw and thus the meat yield is higher [12,66]. There is less sexual dimorphism in black crabs [67], and on Eleuthera Island, where this species is predominant, females were preferred because of the higher fat levels and eggs. In other crab species where the entire body is consumed, females are also preferred because they have a higher fat content in both the gonads and hepatopancreas, which imparts a “sweet umami or buttery” taste [61,68,69].

### 4.2. General Perceptions

Land crab populations around the world are under threat due to habitat destruction, climate change, pollution, and overharvesting [23,27,70]. The same appears to be occurring in The Bahamas, with people reporting a decline in crab numbers, especially for the white land crab. In general, across the islands, the main perceived threats were urbanisation/development and overharvesting.

Anthropogenic habitat alteration and destruction is recognised as the single largest threat to animal diversity [71,72,73]. Here too, land development and urbanisation was perceived as the greatest threat to land crabs in The Bahamas. On New Providence, which is the most developed of the Bahamian islands, urbanisation and development was the only threat that was essentially considered important. This shows that local people working in the environment have an ecological understanding of threats to animals, having witnessed changes over prolonged periods of time [74,75]. Urbanisation and development does not need to take place on a large scale to have noticeable effects: habitat fragmentation, where large habitats are split into smaller isolated pockets, can have devastating impacts [73]. In The Bahamas, forests are cleared for homes or resorts, with access roads constructed into areas that were once largely inaccessible. Because land crabs exhibit a high site fidelity, this can reduce effective foraging areas and put them at risk when crossing roads [27,76], where mortality is substantially increased [77,78]. Exposure in open areas also leaves them more vulnerable to predation and desiccation [20,67]. On Great Exuma Island, two people reported that catches of black land crab had improved because land clearance and new roads made areas easier to access, with fewer places for crabs to hide. This represents a short-term accessibility benefit that contrasts with longer-term ecological risks.

The second most perceived reason (to which nearly a third of respondents strongly agreed) for the decline in crab numbers was overharvesting (Table 3). This is a universal problem for all fished species [79,80]. The idea that crab numbers were declining due to overharvest was not fully supported by some of the feedback: a number of respondents suggested fewer people were crabbing because they had to work, or the youth were less interested in crabbing. However, in recent years, social media has been used to advertise the breeding migration, leading to a higher harvest of females with eggs [19]. Increased tourism (annual crab festival and food tourism) has also led to an increased demand for crabs. There are reports of 400 white crabs being caught each night [19]. We found that although the average reported numbers were somewhat lower and more variable, in a few cases, people stated that they could collect over 700 crabs in one night, although this high number was not typical. Therefore, although fewer people are crabbing, those that do go are investing more effort for monetary incentives rather than sustenance. White crab numbers have also declined in other areas of the Caribbean, with overharvesting stated as being the main reason for the decline and the lack of bigger crabs [21,27,60].

When specifically asked about changes in the white crab population, the majority of respondents indicated a significant drop in numbers, with very few people reporting an increase on any island. These survey results line up with a 90% documented decline in white crabs on Andros during the past 40 years [28,29], most likely due to overharvesting [19]. While the majority of people thought that the number of black crabs had also declined, a greater percentage reported no change or even an increase in numbers compared with white crabs. The decreased concern for black crabs appeared to be related to the fact that crabbers reported seeing large numbers of young/baby crabs on the roads and edges of the forest. However, the smaller crabs that they assumed were juvenile black crabs were more certainly mature red crabs that had been misidentified; this species is much more common than the black crab [10]. Most of the crabbers we talked to were unaware that they are a separate species and even recently assigned to a different genus from black crabs based upon slight morphological differences and new molecular evidence (referred to as *Hartnollius lateralis* [10]). This highlights the need for caution when incorporating LEK with current Western scientific knowledge [81].

While development and overharvesting emerged as the primary threats, with some variation in concerns between white and black crabs, more specific questioning revealed notable differences among islands. Urbanisation, as stated above, was the only real threat reported on New Providence. Forest fires were specifically mentioned on Andros, most of these being quite recent. These fires have not only affected the crabs, but have destroyed critical habitats for other species like the endangered Bahamian oriole [82]. Rodriguez-Fourquet et al. [27] stated that fires were even sometimes started illegally by crabbers to gain access to the area and force the crabs out of their burrows. Although invasive species were not regarded as a particular problem across islands (Table 3), people in northern Eleuthera were concerned about the effect of invasive raccoons on crab populations. Social media posts from Eleuthera, Abaco, and Grand Bahamas give the perception that raccoons are eating black land crabs and causing a significant reduction in numbers (Coastal Angler Magazine, 2012; Facebook). Ecological impacts of invasive raccoons have been verified in other areas of the world, where they have decimated native animal populations including a number of important crustacean species [83,84,85]. Finally, Haitian immigrants were perceived as problematic on Andros and Eleuthera, indicating underlying social tensions. These problems have been documented as far back as the early 1960s, and by the 1990s Haitians were blamed for “every social and medical ill befalling The Bahamas” [86,87,88]. In particular, mass migration, documented or otherwise, does pose threats to food security [89,90].

Although land crabs are a significant resource in current day Bahamas, they were also an important food source in pre-colonial times. On the small Bahamian island of San Salvador, the excavation of middens has shown that the Lucayan people shifted from a crab culture to a shell culture, harvesting molluscs from the oceans when the land crabs became scarce [91]. Similar patterns have been seen in pre-colonial times on other Caribbean Islands where people turn to the sea as terrestrial-based food resources dwindle [92,93,94]. This shows that land crabs are not a finite resource and are prone to overharvest, even in pre-colonial times.

A little over a third (37%) of people interviewed reported seeing baby crabs (megalopa) returning from the sea. Mass recruitment events are rare in the Caribbean as they only happen on certain tides and heavy rains [95]. For black land crabs, recruitment events may only occur every 5–6 years [6,26]. Although we presented photos of the megalopa stage, recruitment events are sporadic [95], and most crabbers were unaware that red crabs constitute a separate species. Therefore, we cannot rule out the possibility that the crabbers were actually witnessing small red crabs, which are known to become active en masse in coastal areas following rainfall events [39].

Studies on insular gecarcinid crabs have highlighted the lack of data on first instar and juvenile stages [46,96] to the degree that they have sometimes been referred to as the lost years [5]. The scarcity of small crabs could be due to different habitat choices (compared with adults), but if recruitment only occurs every few years, then certain age-size classes would be largely absent [27]. One respondent said that the baby black crabs live in burrows with adults, and even juveniles remain near the burrow. This observation has been confirmed in a closely related species *Johngarthia lagostoma* just recently [95], highlighting the importance of LEK, preceding and confirming scientific discovery.

### 4.3. Black Land Crab Biology

Typically much less is known about black land crabs compared with white land crabs [10,19,20]. The preferred habitat of black land crabs in The Bahamas is inland broadleaf limestone forest, also locally referred to as “blackland” coppice. The thick loamy soils and karst topography hold moisture, which form cool humid microhabitats, while fallen leaves provide adequate nutrition for crabs [20]. When asked why the forest was important for black crabs, the majority of responses simply stated that forests were the crabs’ “home” or “habitat”, without elaboration. While these are basic responses, they still show a recognition of crab-forest dependence and could be relevant for conservation framing. Those that did provide details mentioned protection from predators and the environment, suggesting an understanding of predator–prey relationships and the protective function of forests.

When asked for specific areas of the forest where black crabs were found, a clear majority of people mentioned specific structures: under logs, rocks, and leaves and in rock crevices and holes. These observations align with previous research showing that black crabs are incidental burrowers that are more likely to seek cool, moist microhabitats beneath objects or within tree root systems [15,97]. This is in contrast to red crabs and white crabs, which are burrow dwellers, constructing their refuges along edges of the forest or mangrove swamps, respectively [2,15]. Specific feedback of the prevalence of black crabs around sinkholes suggests species-specific habitat knowledge. The sinkholes are considerably cooler than the surrounding forest floor with a stable high humidity, which provides an important microhabitat for black crabs, especially during the winter hibernation period [20].

Maintaining water balance is critical to black crabs’ ability to survive on land. Standing water is uncommon in The Bahamas because the intermittent precipitation quickly percolates through the limestone bedrock [49]. Black crabs can rehydrate by absorbing water from damp soil via capillary action [20] or drinking dew [59]. However, rainfall does present a vital opportunity to maintain hydration, and crabs become noticeably more active on the surface following a rain event [20]. People clearly knew that rain was a key factor for land crabs, reporting that they emerged after the rains and could even be caught during the daytime. Conversations with seasoned crabbers suggested that crabbing was best without any moonlight; however, the results of the survey did not reflect this, and there was no consensus as to which moon phase the crabs were most active. This is an interesting contrast between LEK and the survey outcomes. The LEK that rainfall was the primary driver aligns with scientific knowledge that rainfall and the time between rain events are known to have a stronger influence on crab activity than the moon phase [20,98]. Because heavy summer rains are intermittent, crabbing is obviously opportunistic, and people will crab after these events irrespective of the moon cycle.

In 2002, the Bahamas National Trust set aside 4000 acres of land for a crab preserve to protect critical habitat for white crabs from development and maintain a route for their spawning migration [29]. We did not obtain any feedback on the perceptions of the preserve on Andros; however, it is important to note that the preserve was set up to protect the crab habitat, not as a no-take zone, and the harvesting pressure is still high in the preserve (LaPilusa, pers comm). When asked about the possibility of a black land crab preserve, the responses were mixed, with no clear support for or against it on each island. The lack of consensus and verbal feedback suggested that tensions existed around access to crabbing grounds, management authority, and resource competition between different communities, which might exacerbate the actual implementation of a black land crab preserve and/or any harvesting regulations ([29]; McGaw et al., unpub. obs.). These are important social factors to consider in any future management planning. Those that did support a preserve suggested possible locales in areas where there was already a high abundance of black crabs.

There was a clear understanding that black crabs are opportunistic omnivores; as such, most respondents listed multiple food sources. Over twenty respondents said “anything”; while this could also be interpreted that crabs are opportunistic feeders, this broad awareness also limits the precision in LEK data, highlighting the need to cross validate with ecological studies. Although most decapod crustaceans are classified as omnivores, by the nature of their habitat, land crabs are primarily vegetarian, eating leaves and fruit [99]. Clearly, the respondents were aware of this, with over 50% people confirming leaves and fruit as being their primary source of nutrition. Two people made statements that “once you have a garden they will come”, showing an understanding of their opportunistic diet [39] but also their ability to become an agricultural pest [100]. A significant percentage of people also listed carrion, faeces, and garbage as part of the diet of black crabs, which has been previously documented [99,101]. The consumption of unpalatable items is the reason that people stated the crabs need to be “cleared” before eating. This clearing is different from prolonged holding and feeding to alter taste profiles (discussed above); rather, it allows for waste to be evacuated, which takes 2–4 days [102]. This is important because in contrast to most marine crabs, where only the muscles of the legs and claws are eaten, the entire body contents of land crabs are consumed. Fewer people listed fungi and insects as important dietary items, observations that have been noted in scientific articles [2,97]. Other crabs were listed as a food source for black crabs. The closely related red crab (*G. lateralis*) exhibits cannibalism when food sources are nitrogen limiting, feeding on smaller individuals to supplement their diet [103]. One might expect that black crabs, which are significantly larger than red crabs and overlap in habitat, might also prey on red crabs as an opportunistic meal [99].

**Figure 8:**
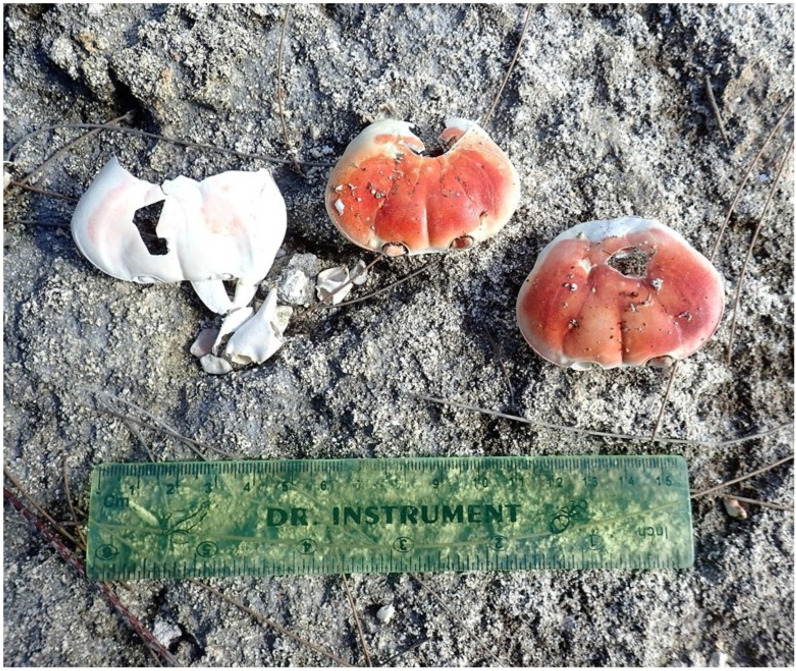
Photograph of the carapaces of *Gecarcinus* species showing indications of predation by night herons (photograph: William Bigelow).

When asked about potential predators of black land crabs, over 70% of respondents listed birds. This is backed-up by the scientific literature, with specific reference to herons as major predators on different species of land crabs [59,104,105,106]. Baine et al. [26] reported that the Yellow Crowned Night Heron (*Nyctanassa violacea*) was the only predator that could take on a black crab when it reached adult size. We have observed Black Crowned Night Herons (*Nycticorax nycticorax*) killing and consuming black crabs. They kill the crab by stabbing it through the carapace with their beak, leaving a crab shell with a characteristic single hole in the back (Figure 8). This strong agreement between LEK and scientific evidence reinforces the reliability of ecological knowledge.

Feral hogs were thought to eat black crabs, and they have decimated other land crab populations on small islands [107]. On the island of Eleuthera, raccoons were regarded as a key predator reducing black crab numbers. Aquatic crustaceans, where available, do form an important part of a raccoon’s diet [84]. Despite the increasing concern in the impacts of invasive raccoons, we do not have any measure of where raccoons prey on black crabs, how often they consume live or dead crabs, and if the crabs gain any refuge in size. In this case, scientific evidence would complement LEK and help inform policy decisions regarding invasive species [43,108].

Ortega-Rubio et al. [105] found land crab remains in cat faeces. Cats were not regarded as predators of black land crabs, even though both feral and pet cats abound in The Bahamas [49]. Approximately 20% of respondents thought that dogs ate black land crabs, stating potcakes (Bahamian dog breed) “chase and bark at crabs”. We do not have any direct evidence of them catching live crabs, but as most of the dogs are free ranging, they are probably eating roadkill [77,78].

Other crabs were listed as potential predators, and cannibalism does occur in other species of land crabs [99,103]. Interestingly, a number of statements suggested that white and black crabs could not coexist, and the white crabs attacked and/or ate black crabs. There is some overlap between white and black crabs in the natural environment, mainly occurring along the fringes of their respective habitats [15,49]. This could be of importance during the breeding migration, when female black crabs leave the forest and migrate to the coast for spawning [17,98]. We could not corroborate any antagonistic interactions between white and black crabs in the scientific literature; here, LEK has provided a unique perspective that warrants further investigation.

## 5. Conclusions

This study demonstrated that there is substantial overlap between traditional and scientific knowledge systems, thus the LEK acquired through generations of hands-on experience and observation can provide valuable insights into species behaviour, habitat use, and ecological processes that complement formal scientific research [29,109,110]. The integration of LEK with scientific data not only fills knowledge gaps, but also enhances the political position of local resource users, making them active participants in conservation efforts rather than passive recipients of top-down management decisions [43,108]. As fisheries and marine resources face increasing pressures from climate change and anthropogenic activities, incorporating local knowledge will become important for developing more effective, culturally appropriate, and sustainable management strategies that benefit both ecological conservation and local communities.

## Figures and Tables

**Table 1:** Importance of land crabs to the economy of Andros, New Providence, and Eleuthera when the respondents were given a choice of not important at all to extremely important. Data shown as percentages, with the number of respondents in parentheses.

	Not at All Important	Slightly Important	Moderately Important	Very Important	Extremely Important
Q20. How important are land crabs to the economy of Andros?	0% (0)	0% 0%	9.4% (3)	18.8% (6)	65.6% (21)
Q20. How important are land crabs to the economy of New Providence?	14.3% (3)	42.9% (9)	42.9% (9)	9.5% (2)	0% (0)
Q20. How important are land crabs to the economy of Eleuthera?	5.8% (3)	34.6% (18)	17.3% (9)	17.3% (9)	25% (13)

**Table 2:** Importance of land crabs to the culture and economy of The Bahamas as a whole. Respondents were given a choice of not important at all to extremely important. Data shown as percentages, with the number of respondents in parentheses. Chi-square analyses and *p* values are shown in the final column.

	Not at All Important	Slightly Important	Moderately Important	Very Important	Extremely Important	Chi-Square
Q19. How important are land crabs to Bahamian culture?	0.9% (1)	1.8% (2)	8.1% (9)	27.9% (31)	61.3% (68)	ꭓ^2^ = 144.45 *p* < 0.001
Q21. How important are land crabs to the Bahamian economy?	11.9% (12)	16.8% (17)	20.8% (21)	17.8% (18)	32.7% (33)	ꭓ^2^ = 14.70 *p* = 0.005

**Table 3:** Feedback on potential threats to land crabs across The Bahamas. Respondents were given a choice as whether they disagreed or agreed with the statements. Data shown as percentages, with the number of respondents in parentheses. Chi-square analyses and *p* values are shown in the final column.

Q. 25	Strongly Disagree	Disagree	Neither Agree nor Disagree	Agree	Strongly Agree	Don’t Know	Chi-Square
a. Climate change is likely to harm the land crab population.	5% (4)	12.5% (10)	23.8% (19)	30% (24)	20% (16)	8.8% (7)	ꭓ^2^ = 16.66 *p* = 0.002
b. There will be less crabs in the future because of overharvesting.	7.5% (6)	15% (12)	17.5% (14)	27.5% (22)	32.5% (26)	0% (0)	ꭓ^2^ = 16.0 *p* = 0.003
c. Land redevelopment: clearing forests building or farming will lead to less crabs in the future.	2.5% (2)	6.3% (5)	3.8% (3)	38.8% (31)	48.8% (39)	0% (0)	ꭓ^2^ = 77.50 *p* < 0.001
d. I’m concerned invasive species will affect land crab populations.	10% (8)	21.3% (17)	16.3% (13)	23.8% (19)	18.8% (15)	10% (8)	ꭓ^2^ = 4.94 *p* = 0.323
e. Overall, I am concerned there will be fewer land crabs in the future.	7.5% (6)	16.3% (13)	16.3% (13)	28.8% (23)	27.5% (22)	3.8% (3)	ꭓ^2^ = 13.07 *p* = 0.019

## Data Availability

The authors can make data available upon request and at their discretion.

## Supplementary Materials

The following supporting information can be downloaded at: https://www.mdpi.com/article/10.3390/ani15202941/s1, Supplemental Data S1: Survey used to assess the responses of the land crabbers. Not all questions and demographics gathered were used in this study; those that were used are highlighted in yellow. Supplemental Data S2: Semantic thematic analysis of questions that had open-ended responses. If people volunteered additional information for other questions, it was usually added into the open-ended questions that most closely addressed this topic.
