# Supplementary material for: Harvesting Practices and Local Ecological Knowledge (LEK) of Bahamian Land Crabs: Bridging Gaps Between Traditional and Scientific Knowledge
Source: Animals (Basel). 2025 Oct 10;15(20):2941. doi: 10.3390/ani15202941 (PMC12560874; doi:10.3390/ani15202941)
Supplement: Supplementary file 1 [file animals-15-02941-s001.zip › animals-3887618-supplementary.pdf]

Land Crab Fishery Survey : Supplementary Data 1

Revised: June 2024

Interviewer Name	Date (DD/MM/YY)	Town	Interview Code

Start Time	Hour		Minute	
Time Interview Started [Interviewer: Enter hour and minute, use 24hr. clock]				

Participant consent read out and confirmed (circle)? Yes or No

Demographics

This is just to get some basic background information about you

**1. Respondent's Sex [DO NOT ASK THEM—PLEASE FILL OUT YOURSELF]**	
**Male**	(1)
**Female**	(2)

**2. How old are you?**	

**3. Are you a permanent resident of the Bahamas?**	(1) Yes	(2) No **[GO TO 3.1]**
**3.1 [IF NO] Where do you permanently reside?**		

**4. Which Island do you currently live on?**	

**5. What is your highest level of education? [DO NOT READ CHOICES/ANSWERS]**	
No formal schooling	(1)
Primary school	(2)
Some secondary school	(3)
Secondary school	(4)
Post-secondary qualifications, e.g. a diploma or associates degree	(5)
Some University	(6)
Completed College / University	(7)
Undergraduate degree completed	(8)
Post-graduate degree	(9)

**6. What is your employment status?**

(0) Not Employed	(1) Part Time **[GO TO 6.1]**	(2) Full Time **[GO TO 6.1]**
**6.1. [IF 1 OR 2] What Industries [CAN BE GENERAL DESCRIPTION]?**		

Land Crab Fishery: Practices

Here we will ask about general information - if you go out, or have ever gone out catching crabs

7. Have you been crabbing before?[CIRCLE]
(1) Yes (2) No **[IF NO GO TO QUESTION 19]**

**8. In which season do/did you go crabbing?**			
(1) Winter (Dry)	(2) Summer (Wet)	(3) Both	(999) Don't know **[DO NOT READ]**

**9. How frequently do you go crabbing during those season(s)?**					
(1) Daily	(2) Weekly	(3) Monthly	(4) A few times a year or less	(5) Other **[GO TO 9.1]**	(999) Don't know **[DO NOT READ]**
**9.1. [IF OTHER, PLEASE RECORD ANSWER]**					

**10. Where or how do you catch land crabs? [READ OUT CHOICES & CHECK ALL THAT APPLY]**			
(1) On the side of the road	(2) Deep in the bush	(3) Dig for crabs	(4) Other **[GO TO 10.1]**
**10.1. [IF OTHER, RECORD ANSWER]**			

**11. Who do you normally go crabbing with? [READ OUT CHOICES & CHECK ALL THAT APPLY]**				
Myself (1)	Family (2)	Friends (3)	Team / Partner (4)	Other (5) **[GO TO 11.1]**
**11.1. [IF OTHER, RECORD ANSWER]**				

**12. How many hours do you typically go crabbing on a given night?**

13. On a good night, how many crabs do you catch?

14. What kind of land crabs do you catch? [CIRCLE EACH THAT APPLY]		
**(1)**White Crab 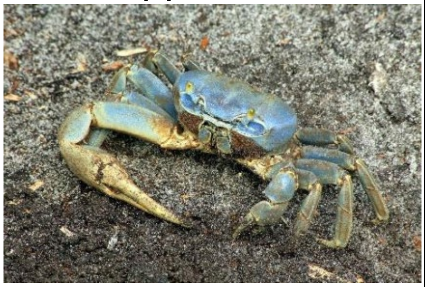	(2)Black Crab 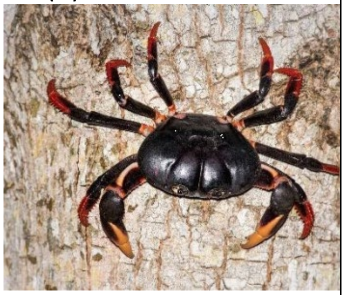	(3) Red/Soldier Crab 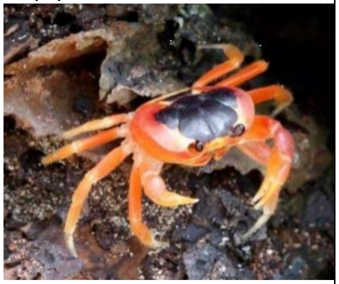

15. Do you prefer to catch white land crabs or black land crabs or do you have no preference?			
White **[GO TO 15.1]**	Black **[GO TO 15.1]**	(3) No preference	(999) Don't know **[DO NOT READ]**
**15.1. Why do you prefer _______?**			

16. Do you prefer to catch male crabs or female crabs or do you have no preference?			
(1) Male **[GO TO 16.1]**	(2) Female **[GO TO 16.1]**	(3) No preference	(999) Don't know **[DO NOT READ]**
**16.1. Why do you prefer _______?**			

17. What do you do with the crabs that you catch? [READ OUT CHOICES/ANSWERS & CHECK ALL THAT APPLY]					
(1) Personal Consumption	(2) Share with family and friends	(3) Sell Locally	(4) Sell to other Islands	(5) Other [GO TO 17.1]	(999) Don't know **[DO NOT READ]**

**17.1 [IF OTHER, LIST]**

**18. On a scale from 1-5, with 1 being not at all important and 5 being extremely important, how important are land crabs to you financially?** [IF THE RESPONDENT HAS TROUBLE GIVING A NUMBER, PLEASE PROVIDE THEM WITH THE WORD EQUIVALENTS: **1=Not at all important; 2=Slightly important; 3=Moderately important; 4=Very important; 5=Extremely important.]_____**

Land Crab Fishery: General Perceptions and Attitudes

Here we will ask you about your perceptions about land crabs, you do not have had to have been crabbing to answer these questions.

**19. On a scale of "1-5", with 1 being not at all important and 5 being extremely important, how important are land crabs to your culture?** [PLEASE PROVIDE THE WORD EQUIVALENTS IF NEEDED: **1=Not at all important; 2=Slightly important; 3=Moderately important; 4=Very important; 5=Extremely important.]**	

**20. On the same scale, how important are land crabs to your local economy (this town or island)?**	

**21. On the same scale, how important are land crabs to the economy of The Bahamas?**	

**22. How often do you eat land crab during the crabbing season?**					
(1) Daily	(2) Weekly	(3) Monthly	(4) A few times a year	(5) Other **[GO TO 22.1]**	(999) Don't know **[DO NOT READ]**
**22.1. [IF OTHER, RECORD ANSWER]**					

**23. Do you think there are more or less white land crabs on this Island [WHERE INTERVIEW TAKING PLACE] than there were five to ten years ago, or has there been no change?**			
Less **(1)[GO TO 23.1]**	No change (2)	More **(1)[GO TO 23.1]**	(999) Don't know **[DO NOT READ]**
**23.1 [IF INDICATED CHANGE] Why do you think so?**			

24. Do you think there are more or less black land crabs on this Island [WHERE INTERVIEW TAKING PLACE] than there were five to ten years ago, or has there been no change?			
Less<br>**(1)[GO TO 24.1]**	No change<br>(2)	More<br>(3) **[GO TO 24.1]**	(999) Don't know **[DO NOT READ]**
**24.1 [IF INDICATED CHANGE] Why do you think so?**			

**25. For each of the statements I read out, could you please tell me how strongly you agree or disagree with each? Do you strongly agree, agree, disagree or disagree strongly?**							
		Strongly Disagree	Disagree	Neither agree nor disagree	Agree	Strongly Agree	Don't know (DNR)
a	Land crabs should be preserved for future generations	1	2	3	4	5	999
b	Climate change is likely to harm the land crab population.	1	2	3	4	5	999
c	There will be less crabs in the future because of over harvesting, that is people taking too many crabs.	1	2	3	4	5	999
d	Land redevelopment, such as clearing forests for buildings of farming, will lead to less crabs in the future.	1	2	3	4	5	999
e	I'm concerned invasive species will affect land crab populations.	1	2	3	4	5	999
f	Overall, I am concerned there will be fewer land crabs in the future.	1	2	3	4	5	999

Land Crab Fishery: Potential Regulations

Here we want to find out about any rules or laws concerning catching land crabs

**26. Are there any rules/laws for harvesting land crabs in the Bahamas?**		
(1) Yes	(2) No	(999) Don't Know **[DO NOT READ]**
**26.1. Do you think there should be rules or laws for harvesting land crabs?**		
(1) Yes **[CONTINUE TO 26.2]**	(2) No **[SKIP TO 27]**	(999) Don't Know **[DO NOT READ]**
**26.2. [IF YES] What rules would support? [READ OUT CHOICES AND CHECK ALL THAT APPLY]**		

Catch limits (1)	Size limits (2)	Not retaining females with eggs (3)	Limited crabbing season. (4)	Other (5) [GO TO 26.3]	Don't know [Do not read] (999)

26.3 [IF OTHER, RECORD ANSWER]:

26.4. Who do you think should enforce these rules? [READ OUT CHOICES/ANSWERS & CHECK ALL THAT APPLY]

Fishery Officers/ Game wardens (1)	The Police (2)	Politicians (3)	People in the Local Community (4)	Other (5) **[GO TO 26.5]**	Don't know **[DO NOT READ]** (999)

26.5 [IF OTHER, RECORD ANSWER]

27. Generally speaking, how much do you trust the Government or Fisheries Departments to do what is right for the locals and land crab fishery?

No trust at all (1)	A little bit of trust (2)	A fair amount of trust (3)	A great deal of trust (4)	Don't know **[DO NOT READ]** (999)

28. Now, I will again read out a couple of statements. Could you please tell me how strongly you agree or disagree with each? Do you strongly agree, agree, disagree or disagree strongly?

	Strongly Disagree	Disagree	Neither agree nor disagree	Agree	Strongly Agree	Don't know (DNR)
A. Most people can be trusted.	1	2	3	4	5	999
B. I trust other crabbers in my area to catch crabs in a way that leaves enough for the future.	1	2	3	4	5	999

29. Please tell me how often you, personally, have gotten together with others in the community to try and address an issue or solve a problem in your town during the last five years.				
Never	Only once	Sometimes	Often	Don't know *[Do not read]*
0	1	2	3	999

**30. Other than church, are you a member of any community associations or organizations?**	
No	0
Yes **[IF YES, LIST THE ORGANIZATIONS/ASSOCIATIONS]**	1
Don't know ***[DO NOT READ]***	999

**31) In your opinion, who should be allowed to harvest land crabs around here?**			
	No	Yes	Don't know ***[DO NOT READ]***
**A.** Local residents of the town	0	1	999
**B.** Residents of this island	0	1	999
**C.** Bahamian citizens from other islands	0	1	999
**D.** Bahamian commercial operators	0	1	999
**E.** Tourists and other non-residents	0	1	999

Black Crab (only): Local Knowledge

Here we are only asking about the black crab (not white or red crabs), we want you to help us learn about black land crabs

**32. Where do you find black crabs in the forest/bush? [Probe for specific areas: e.g. in burrows, under logs, sinkholes, under leaves]**

**33. During what season do you see the most black crabs running?**		
Rainy Season / Summer (1)	Dry Season / Winter (2)	Don't know (999) [Do not read]

**34. During that time, do you see the most black crabs running when there is a full moon or when there is a new moon?**		
Full Moon (1)	New Moon (2)	Don't know (999) **[DON'T READ]**

35. What do you think black crabs eat? [DO NOT READ CHOICES/ANSWERS, CHECK ALL THAT ARE MENTIONED BY THE RESONDENT]								
Leaves/ plants (1)	Dead Animals (2)	Fruit (3)	Feces (4)	Fungus (5)	Garbage (6)	Other crabs (7)	Other, please specify (8) **[GO TO 35.1]**	Don't know (999) **[DON'T READ]**
**35.1 Other:**								

36. Have you ever seen baby land crabs returning from the sea? [INTERVIEWER: SHOW PICTURE]		
(1) Yes	(2) No	(999) Don't know **[DON'T READ]**
**36.1 [IF YES] When was the last time and where you recall seeing this?**		

37. Other than people, what else eats black crabs? [DO NOT READ CHOICES/ANSWERS, CHECK ALL THAT ARE MENTIONED BY THE RESONDENT]						
Birds (1)	Wild boar/feral pigs (2)	Snakes (3)	Racoons (4)	Dogs/cats (5)	Other (6) [GO TO 37.1]	Don't know (999) **[DNR]**
**37.1 Other:**						

38. Do you think the bush/forest is important for black crabs?		
(1) Yes **[GO TO 38.1]**	(2) No	(999) Don't know **[DNR]**
**38.1. [IF YES] Why?**		

**39. Do you think there should be an area of forest on the Island that should be a black land crab preserve where they cannot be harvested?**		
(1) Yes<br>**[GO TO 39.1]**	(2) No	(999) Don't know **[DNR]**
**39.1 [IF YES] Any ideas for location and why?**		

Follow Up Questions

**40. Are there any other things about land crabs that you would like to share with us**

**41. How can scientists best support the continued crab harvest /crab harvesters?**

Thank you very much for taking the time!

End Interview

End Time	Hour		Minute	
**Time Interview Ended [Interviewer: Enter hour and minute, use 24hr. clock]**				

**Length. Duration of interview in minutes**			

Supplementary Data 2: Semantic Thematic Analysis

Q15: Do you prefer to catch white crabs or black crabs?

White Crabs: Preferred Characteristics

Primary Reasons for Preference

Theme	Frequency	Representative Quotes
Superior Taste	Very High	"Taste better", "I prefer the taste", "Better taste"
Size/Meat Yield	High	"Bigger and more meat", "The white crabs have more meat", "Larger crabs"
Economic Value	Moderate	"Can be sold for a higher price", "More profitable", "Sells for a better price"
Ease of Catching	Moderate	"Easier to catch", "Black crabs are harder to catch and fight back"
Abundance/Availability	Moderate	"More plentiful", "Only see the white crabs", "More abundant"
Familiarity	Low	"These are the ones I grew up catching", "Grew up catching these ones"
Durability in Captivity	Low	"Keep white ones for weeks", "Seem to last longer in pens"

Culinary Observations

Theme	Representative Quotes
Fat Content	"More fat so tastes better", "More fat - especially female crabs"
Standalone Dish	"White crab meat can be eaten on its own", "Taste better on their own - black crab you have to eat with rice"
Versatility	"Better size, more recipes for cooking", "Better dishes/recipes"

Black Crabs: Preferred Characteristics

Primary Reasons for Preference

Theme	Frequency	Representative Quotes
Superior Taste	Very High	"Taste better", "Sweeter taste", "Better taste when cooked"
Safety in Handling	Low	"Smaller claws making it less risky to catch"
Habitat Quality	Low	"Found in cleaner areas"
Catching Experience	Low	"Don't run away as much when trying to be caught", "Rarer (harder to find) making it more of a challenge"
Regional Availability	Low	"Black crabs are more common in New Providence"

Culinary Observations

Theme	Representative Quotes
Companion	"Black crab is usually eaten with something else", "Crab and Rice is causing too

Theme	Representative Quotes
Foods	many people to crab, its just too good"

Red Crabs: Notable Characteristics

Primary Uses (Not Widely Preferred for Eating)

Theme	Frequency	Representative Quotes
Bait Usage	High	"Red/Soldier crabs are used as bait (fishing)", "Red/Soldier crab used as bait, known as Gallen crab"
Size Limitation	Moderate	"Too small to eat", "Baby crabs"
Alternative Names	High	"Called diddler crabs", "Soldier crabs also called joombies", "Jumbee - Baby crabs"
Occasional Consumption	Low	"Sometimes eaten", "Grandma would make them into a stew"

Cultural & Economic Significance

Cultural Practices

Theme	Representative Quotes
Holiday Traditions	"Crabs play an important part of fathers day for cooking", "Crabs important for fathers day"
Regional Preferences	"Both sell; up north they like white crabs, in the south people like black crabs"

Economic Activities

Theme	Representative Quotes
Market Value	"White crabs are more profitable", "Black crabs more marketable"
Value-Added Practices	"Better to pen and fatten", "Can be used for food and even for fishing"

Comparison Analysis

White vs. Black Crabs

Feature	White Crabs	Black Crabs
Taste Profile	Generally preferred for standalone eating	Described as "sweeter," often paired with rice
Size	Consistently described as larger	Smaller, less meat
Handling Difficulty	Described as more aggressive, "feisty"	Easier to handle, smaller claws
Culinary Versatility	More recipes, can be eaten alone	Often associated specifically with rice dishes
Economic Value	Higher selling price mentioned multiple times	Less mentioned for economic value
Abundance	Described as more plentiful	Described as rarer, more of a challenge to find

Red Crabs

Red crabs stand apart as primarily utilitarian rather than culinary, with their main value being as fishing bait. They are significantly smaller than both white and black crabs, with specialized local names (diddler, joombies, Gallen) indicating their cultural recognition despite limited food value.

Additional Cultural Insights

The responses reveal a rich cultural context around land crabs, including:

1. Regional identity markers - Different regions prefer different types of crabs
2. Intergenerational knowledge transfer - References to growing up catching specific types
3. Seasonal/holiday significance - Special importance for Father's Day celebrations
4. Specialized preparation methods - Knowledge about cleaning, fattening, and cooking techniques
5. Economic ecosystem - Different market values and selling practices
6. Environmental concerns - One note about overharvesting: "Crab and Rice is causing too many people to crab"

This analysis suggests land crabs are not merely a food source but represent a significant cultural practice with economic implications, specialized knowledge systems, and regional variations in preference and preparation.

Thematic Analysis

White Crabs (51 responses)

1. **Taste & Culinary Qualities (37.3%)**
   - Better taste/flavor: 29.4% (15 responses)
   - More fat: 5.9% (3 responses)
   - Better for recipes: 2.0% (1 response)
2. **Size & Meat Yield (19.6%)**
   - Larger size: 7.8% (4 responses)
   - More meat: 11.8% (6 responses)
3. **Economic Value (13.7%)**
   - Better price/more profitable: 9.8% (5 responses)
   - More marketable: 3.9% (2 responses)
4. **Ease of Harvest (17.6%)**
   - Easier to catch: 11.8% (6 responses)

- Less hostile/less feisty: 5.9% (3 responses)

5. **Availability (11.8%)**
    - More abundant/plentiful: 9.8% (5 responses)
    - More commonly seen: 2.0% (1 response)
6. **Cultural Significance (7.8%)**
    - Traditional/grew up catching them: 5.9% (3 responses)
    - Important for Father's Day: 3.9% (2 responses)
7. **Practical Advantages (5.9%)**
    - Can be kept longer: 3.9% (2 responses)
    - Versatility (food and fishing): 2.0% (1 response)
8. **Serving Preferences (5.9%)**
    - Can be eaten alone: 5.9% (3 responses)

Black Crabs (12 responses)

1. **Taste (66.7%)**
    - Better/sweeter taste: 58.3% (7 responses)
    - Better when cooked: 8.3% (1 response)
2. **Ease of Harvest (16.7%)**
    - Smaller claws/less risky: 8.3% (1 response)
    - Don't run away as much: 8.3% (1 response)
3. **Environmental Factors (8.3%)**
    - Found in cleaner areas: 8.3% (1 response)
4. **Challenge/Rarity Value (8.3%)**
    - Rarer/more challenging to find: 8.3% (1 response)
5. **Regional Availability (8.3%)**
    - More common in New Providence: 8.3% (1 response)
6. **Culinary Context (16.7%)**
    - Usually eaten with something else/with rice: 16.7% (2 responses)
7. **Market Value (8.3%)**

- More marketable: 8.3% (1 response)

Red Crabs (10 responses)

1. **Nomenclature/Local Names (70%)**
    - Called "diddler" crabs/diddlas: 20% (2 responses)
    - Called "soldier" crabs: 30% (3 responses)
    - Called "joombies"/"jumbee": 30% (3 responses)
2. **Utilitarian Purpose (40%)**
    - Used as bait for fishing: 40% (4 responses)
3. **Size Characteristics (20%)**
    - Smaller size: 20% (2 responses)
4. **Culinary Usage (20%)**
    - Sometimes eaten/made into stew: 10% (1 response)
    - Too small to eat: 10% (1 response)
5. **Taste (10%)**
    - Sweet taste when cooked: 10% (1 response)
6. **Habitat (10%)**
    - Found in rocky areas: 10% (1 response)

Comparison: White vs. Black Crabs

Similarities:

- Both valued for their taste, though described differently
- Both harvested for consumption and sale
- Both have specific culinary contexts

Key Differences:

1. **Taste Characterization:**
    - White crabs: Generally "better tasting" without specific flavor descriptors
    - Black crabs: Specifically described as "sweeter"
2. **Culinary Context:**
    - White crabs: Can be eaten alone, versatile in recipes

    - Black crabs: Often paired with rice or other foods
3. **Physical Attributes:**
    - White crabs: Larger, more meat, more fat
    - Black crabs: Smaller claws mentioned as advantage for catching
4. **Harvesting Difficulty:**
    - White crabs: Easier to catch, less aggressive
    - Black crabs: Seen as more challenging to catch but less likely to flee
5. **Market Value:**
    - White crabs: Consistently described as more profitable
    - Black crabs: One mention of being "more marketable"
6. **Availability:**
    - White crabs: More abundant overall
    - Black crabs: More common in specific areas (New Providence)

Cultural Insights & Unusual Responses

1. **Father's Day Connection:** Multiple respondents mentioned white crabs' importance to Father's Day celebrations, suggesting a cultural tradition around crab harvesting and consumption for this holiday.
2. **Regional Preferences:** Interesting note about regional differences: "up north they like white crabs, in the south people like black crabs," indicating geographic variation in taste preferences.
3. **"Jumbee"/"Joombies":** The local name for red/soldier crabs has spiritual connotations, as "jumbee" typically refers to spirits or ghosts in Caribbean folklore, possibly reflecting these crabs' quick, erratic movements.
4. **Concern About Overharvesting:** One respondent noted "Crab and Rice is causing too many people to crab, its just too good," suggesting awareness of potential sustainability issues due to culinary popularity.
5. **Generational Knowledge:** References to growing up catching specific types and grandmothers' recipes indicate intergenerational knowledge transfer about crab harvesting and preparation.
6. **Preservation Methods:** Knowledge about penning and fattening crabs, and observations about which types "last longer" suggests traditional preservation techniques.
7. **Ecological Knowledge:** Comments about habitat (rocky areas for red crabs) and behavior (black crabs don't run away as much) demonstrate local ecological knowledge.

8. **Female Crabs:** Specific mention of female white crabs having more fat suggests gender-based selection criteria among experienced harvesters.

Q16. Do you prefer to catch male or female crabs?

Female Preference Analysis

1. **Egg-Related Preferences (75.0%)**
    - Eggs valued for consumption: 50.0% (18 responses)
    - Eggs specifically valued for cooking/recipes: 22.2% (8 responses)
    - "Cheesy effect" from eggs: 2.8% (1 response)
2. **Fat Content (58.3%)**
    - General fat preference: 41.7% (15 responses)
    - Fat specifically valued for cooking: 16.7% (6 responses)
3. **Flavor Characteristics (8.3%)**
    - Sweeter taste: 2.8% (1 response)
    - More flavorful: 2.8% (1 response)
    - Better taste in cooking: 2.8% (1 response)
4. **Husbandry Advantages (5.6%)**
    - Easier to fatten: 5.6% (2 responses)
5. **Behavioral Characteristics (5.6%)**
    - Less aggressive when catching: 5.6% (2 responses)
6. **Conservation Awareness (2.8%)**
    - Avoid when spawning: 2.8% (1 response)
7. **Health Considerations (5.6%)**
    - High cholesterol content (negative): 5.6% (2 responses)

Male Preference Analysis

1. **Size Advantages (35.7%)**
    - Generally bigger: 21.4% (3 responses)
    - More meat content: 14.3% (2 responses)
2. **Absence of Eggs (50.0%)**
    - Dislike of eggs (avoidance): 42.9% (6 responses)
    - Simple preference for no eggs: 7.1% (1 response)

3. **Husbandry Advantages (7.1%)**
    - Can be kept easier: 7.1% (1 response)
4. **Behavioral Characteristics (14.3%)**
    - Not as aggressive: 7.1% (1 response)
    - "Bigger biters" (possibly referring to claws/defensive capability): 7.1% (1 response)
5. **Flavor Considerations (14.3%)**
    - Taste equivalence (with more meat benefit): 7.1% (1 response)
    - Avoidance of excessive fat: 7.1% (1 response)

Comparison: Female vs. Male Preferences

Key Differences:

1. **Primary Value Proposition:**
    - Female preference is primarily driven by presence of desirable attributes (eggs and fat)
    - Male preference is largely driven by absence of undesired attributes (no eggs) and size advantages
2. **Culinary Applications:**
    - Females: Extensive reference to cooking applications (28% mentioned cooking specifically)
    - Males: Very minimal reference to cooking applications (0 direct cooking references)
3. **Size/Meat Considerations:**
    - Females: No mentions of size as an advantage
    - Males: Size and meat content represent primary positive attributes (35.7%)
4. **Fat Perception:**
    - Females: Fat viewed positively by most (58.3%)
    - Males: Fat sometimes viewed negatively as excessive (7.1%)
5. **Flavor Descriptors:**
    - Females: Some specific flavor attributes mentioned (sweeter, more flavorful)
    - Males: Minimal flavor descriptions, only comparative statements

Shared Elements:

1. **Behavioral Characteristics:**
   - Both groups mentioned aggression levels, suggesting this is an important consideration in selection
2. **Pragmatic Approach:**
   - Some respondents in both categories indicated flexibility ("We take anything but...")

Local Ecological Knowledge Insights

1. **Reproductive Biology Knowledge**
   - Clear understanding of sexual dimorphism and recognition of egg-bearing females
   - Awareness of spawning cycles ("avoid when spawning")
   - Recognition of fat distribution differences between sexes
2. **Husbandry Practices**
   - Differential fattening potential ("Easier to fatten" for females)
   - Keeping conditions ("Can be kept easier" for males)
   - Implied differential handling due to aggression levels
3. **Nutritional Understanding**
   - Recognition of cholesterol content in crab meat
   - Understanding of fat distribution and its relationship to flavor
   - Knowledge of how eggs contribute to overall flavor profile
4. **Behavioral Observations**
   - Differential aggression levels noted between sexes
   - "Bigger biters" suggests observation of defensive behavior differences

Cultural Practice Insights

1. **Culinary Traditions**
   - Specific cooking applications for eggs ("cheesy effect")
   - Fat content valued differently based on cooking methods
   - Implied recipes that specifically utilize female crabs with eggs

2. **Value Hierarchies**
    - Clear preference patterns (36 responses for females vs. 14 for males)
    - Economic implications (females potentially more valuable due to eggs)
    - Complex taste preferences that go beyond simple flavor to include texture and richness
3. **Knowledge Transmission**
    - Specific terminology ("cheesy effect") suggesting community-based knowledge sharing
    - Consistent patterns in descriptions suggesting cultural consensus about attributes
4. **Health Consciousness**
    - Awareness of cholesterol content reflects modern health considerations integrated with traditional practices
    - Balancing traditional preferences with health concerns

Particularly Notable Findings

1. **Culinary Sophistication:** The specific mention of eggs creating a "cheesy effect" in cooking reveals sophisticated culinary knowledge and specialized preparation techniques.
2. **Reproductive Conservation Awareness:** The comment about avoiding females when spawning suggests some ecological conservation awareness integrated with harvesting practices.
3. **Fat Complexity:** The nuanced view of fat (positive in most female-preference responses but sometimes negative in male-preference responses) suggests contextual valuation of this attribute.
4. **Pragmatic Flexibility:** Several responses indicated situational flexibility despite having preferences, suggesting practical adaptability in harvesting practices.
5. **Husbandry Distinctions:** Different sexes appear to be subject to different management practices in captivity, indicating specialized knowledge of sex-specific needs.

This analysis reveals sophisticated ecological knowledge about crab biology, behavior, and culinary properties that informs gender-specific harvesting and preparation practices, reflecting a rich tradition of local ecological knowledge in Bahamian crab harvesting.

Q17. What do you do with the crabs that you catch?

Primary Activities

1. **Flushing/Cleaning Practices (24.4%)**
   - Flushing crabs before consumption: 12.2% (5 responses)
   - Flushing before selling: 7.3% (3 responses)
   - General cleaning practices: 4.9% (2 responses)
2. **Cultural Significance (24.4%)**
   - Father's Day celebrations: 24.4% (10 responses)
3. **Commercial Activities (17.1%)**
   - Selling practices: 9.8% (4 responses)
   - Pricing information: 7.3% (3 responses)
4. **Pen Management Practices (14.6%)**
   - Feeding practices in pens: 9.8% (4 responses)
   - Keeping/maintenance in pens: 4.9% (2 responses)
5. **Catch Disposition (12.2%)**
   - Catch and release: 7.3% (3 responses)
   - Consumption patterns: 4.9% (2 responses)
6. **Other Uses (7.3%)**
   - Events/competitions: 2.4% (1 response)
   - Preservation (freezing): 2.4% (1 response)
   - Gifting: 2.4% (1 response)

Local Ecological Knowledge Insights

1. **Husbandry Practices**
   - **Specific Feeding Regimens:**
     - Dilly leaves as preferred food: "Crabs like being fed dilly leaves"
     - Sweet food preferences: "Feed them mango in the pens to make them taste sweet"
     - Diet diversification: "Crabs in pens fed rice, grape leaves and coconuts to clean them"

        - Diet duration: "Give them a special diet for two weeks with bread and the mango"
        - Dietary restrictions: "Don't feed cooked food in pens they die faster"
    - **Survival Management:**
        - Heat sensitivity: "The Heat can kill them in the cage, so you must give them water"
        - Longevity in captivity: "You can keep and clean and feed in pens for 2 months"
2. **Supply Chain Knowledge**
    - Source Regions: "Most crabs come from Andros, Cat island, Acklins"
    - Distribution Systems: "Selling crabs via boats happens nightly, order quotas are used by locals (by the dozen)"
    - Batch Processing: "Crabs caught in sets, while some are being flushed the next set is sold"

Cultural Insights

1. **Father's Day Significance**
   The overwhelming association with Father's Day (24.4% of responses) suggests a deeply embedded cultural tradition linking crab consumption specifically to this holiday, representing significant cultural patrimony.
2. **Economic Practices**
    - Price Memory: "Prices raised a lot, a dozen was once $12 versus current $30-40 for a dozen"
    - Tiered Pricing Structure: "Different prices of sale for crabs - regular customer versus restaurant"
3. **Sensory Perceptions**
    - Negative perceptions: "They are nasty, at least in smell"
    - Preference hierarchies: "Not a preferred taste, prefer ocean crabs"
    - Taste modification through diet: Evidence of manipulating flavor through specific feeding regimens
4. **Community Relations**
    - Reciprocity: "Sometimes I'll go out and catch them for other people to sell instead for myself"
    - Gifting Networks: "Some people will freeze their crabs to give to family abroad"

5. **Recreational Value**
    - "Some are used for events, such as the biggest crab contest or a crab race"
    - "Catch and release" practices indicating non-consumptive value

Particularly Interesting Findings

1. **Flavor Engineering:** The practice of feeding crabs specific foods (mango, dilly leaves, grape leaves) to intentionally modify their flavor demonstrates sophisticated ecological knowledge about how a crab's diet affects its taste.
2. **Cultural Calendar:** The strong association with Father's Day suggests crabs play a role in marking time and celebrations in the cultural calendar.
3. **Multi-Stage Processing:** The "flushing" obsession and the system of having different sets at different stages of preparation shows sophisticated processing knowledge.
4. **Price Memory:** The specific recall of historical pricing ($12 versus current $30-40) suggests the commodity has important economic history.
5. **Care Knowledge:** Understanding of fatal conditions (heat) and longevity limits (2 months in captivity) indicates developed husbandry practices.
6. **Diet Contraindications:** Knowledge that "cooked food" causes faster mortality shows trial-and-error learning in crab management.
7. **Transnational Networks:** The practice of freezing crabs to send abroad indicates diaspora connections and the importance of maintaining food traditions across distances.
8. **Competitive Uses:** The mention of crab contests and races shows non-culinary cultural uses that still value the resource.

This analysis reveals that crabs are not merely a food resource but represent a complex nexus of ecological knowledge, economic systems, cultural traditions, and community practices within Bahamian society.

Q23, Do you think there are more or less white crabs on this island than there were five to ten years ago, or has there been no change?

ELEUTHERA

Reasons for Population Decline (28 responses, 80%)

1. **Invasive Species (32.1%)**
   - Raccoon predation: 32.1% (9 responses)
2. **Human Harvest Practices (32.1%)**
   - Over-harvesting (general): 17.9% (5 responses)
   - Digging for crabs: 10.7% (3 responses)
   - Out-of-season harvesting: 3.6% (1 response)
3. **Development & Habitat Loss (28.6%)**
   - General development: 21.4% (6 responses)
   - Resort development specifically: 7.1% (2 responses)
4. **Demographic Attribution (10.7%)**
   - Haitian immigrants specifically: 10.7% (3 responses)
5. **Environmental Factors (3.6%)**
   - Less rainfall: 3.6% (1 response)
6. **Other Factors (7.1%)**
   - Pollution: 3.6% (1 response)
   - Population growth: 3.6% (1 response)

Reasons for Population Increase (7 responses, 20%)

1. **Observational Evidence (57.1%)**
   - Personal sightings: 57.1% (4 responses)
2. **Market Factors (14.3%)**
   - Lower harvest pressure due to market preference: 14.3% (1 response)
3. **Environmental Changes (specific to Exuma) (42.9%)**
   - Land clearing increasing visibility: 14.3% (1 response)
   - Increased rainfall: 14.3% (1 response)

- More people crabbing (detection bias): 14.3% (1 response)

ANDROS

Reasons for Population Decline (21 responses, 95.5%)

1. **Harvest Practices (57.1%)**
    - Targeting spawning crabs: 28.6% (6 responses)
    - Targeting females with eggs: 14.3% (3 responses)
    - Taking young crabs: 9.5% (2 responses)
    - General overharvesting: 4.8% (1 response)
2. **Forest Fires (38.1%)**
    - General fire impacts: 33.3% (7 responses)
    - Specific mechanisms (smoke in burrows): 4.8% (1 response)
3. **Demographic Attribution (14.3%)**
    - "Illegal immigrants": 14.3% (3 responses)
4. **Development (4.8%)**
    - General development: 4.8% (1 response)
5. **Biological Factors (4.8%)**
    - Cross-breeding with other crabs: 4.8% (1 response)

Reasons for Population Increase (1 response, 4.5%)

1. **Habitat Expansion (100%)**
    - More areas for crabs to thrive: 100% (1 response)

NEW PROVIDENCE

Reasons for Population Decline (20 responses, 100%)

1. **Development & Urbanization (75%)**
    - General development/habitat destruction: 40% (8 responses)
    - Human population increase: 20% (4 responses)
    - "More homes for people, less homes for crabs": 5% (1 response)
    - Disturbance factors: 5% (1 response)
    - Port/cruise development: 5% (1 response)

2. **Environmental Factors (10%)**
    - Hurricane impacts: 5% (1 response)
    - Weather/rainfall changes: 5% (1 response)
3. **Harvest Practices (5%)**
    - Harmful digging practices: 5% (1 response)
4. **Predation (5%)**
    - Increased predators: 5% (1 response)
5. **Market Dynamics (5%)**
    - Now mostly imported from other islands: 5% (1 response)

Cross-Island Comparison

Primary Perceived Threats by Island

1. **Eleuthera:**
    - Raccoons (32.1%) and development (28.6%) are nearly equally dominant concerns
    - Unique focus on raccoon predation not emphasized on other islands
    - Specific attribution to Haitian immigrants (10.7%)
2. **Andros:**
    - Harvest practices (57.1%) are the dominant concern
    - Uniquely high focus on reproductive threats (targeting spawning crabs/females with eggs: 42.9%)
    - Forest fires (38.1%) as a major threat unique to Andros
3. **New Providence:**
    - Development and urbanization overwhelmingly dominant (75%)
    - Minimal mention of harvest practices (5%)
    - Only island with 100% perception of decline

Contrast in Population Optimism

1. Eleuthera: 20% of responses perceive population increase
2. Andros: 4.5% of responses perceive population increase
3. New Providence: 0% perceive population increase

Contrast in Attribution Patterns

1. Eleuthera: Blame distributed across wildlife (raccoons), development, and specific human groups
2. Andros: Focus on harvesting behaviors and environmental disasters (fires)
3. New Providence: Overwhelmingly attributed to urbanization and development

Unique Causal Factors by Island

1. **Eleuthera Only:**
   - Raccoon predation as major factor
   - Pollution mentioned
2. **Andros Only:**
   - Forest fires as major threat
   - Cross-breeding with other crabs
   - Specific focus on reproductive harvest practices
3. **New Providence Only:**
   - Hurricane impacts
   - Reference to importation from other islands
   - "More homes for people, less homes for crabs" framing

Cultural Insights

1. **Inter-island Migration Perspective:** The notion that crabs are now "imported from the out islands" to New Providence shows awareness of changing harvest geography.
2. **Demographic Attribution:** Both Eleuthera and Andros respondents specifically attribute some harvest pressure to immigrant populations, revealing potential social tensions around resource use.
3. **Knowledge Transfer:** The response "friends from Andros were telling me" indicates inter-island knowledge networks about environmental conditions.
4. **Traditional Ecological Knowledge:** Specific observations about rainfall patterns and crab abundance demonstrate climate-wildlife connections recognized in local knowledge.
5. **Skepticism of Scientific Assessment:** "There is no change scientists don't look hard enough for them" reveals interesting critique of scientific methodology.
6. **Market Dynamics Understanding:** Recognition that white crabs are "not prized as much as black crab" on Eleuthera shows awareness of market preferences affecting harvest pressure.

7. **Harvest Timing Knowledge:** References to "crabbing out of season" and impacts of "taking spawning crabs" demonstrate awareness of life cycle timing.
8. **Development Critique:** The direct statement "new resorts are killing them off and taking their land" shows recognition of tourism development impacts.
9. **Metaphorical Expression:** "More homes for people, less homes for crabs" uses powerful parallelism to express habitat competition.
10. **Variability Recognition:** "Sometimes it's a good night and sometimes it's a bad night" acknowledges natural fluctuations distinct from long-term trends.

This analysis reveals how perceptions of land crab population changes are highly localized, with each island focusing on different primary threats. Eleuthera's concern with raccoons, Andros's focus on harvest practices and fires, and New Providence's emphasis on urbanization demonstrate how environmental perceptions are shaped by distinct local conditions and experiences even within the same archipelago.

Q24 , Do you think there are more or less black crabs on this island than there were five to ten years ago, or has there been no change?

1.Habitat destruction

Eleuthera*:*

- **Frequency**: 8 mentions
- **Key Themes**:
  - Land development
  - Clearing of forests
  - Habitat transformation
- **Numerical Value**: 47% of responses referenced habitat changes

Andros:

- **Frequency**: 4 mentions
- **Key Themes**:
  - Forest fires
  - Habitat destruction
- **Numerical Value**: 33% of responses mentioned habitat issues

New Providence:

- **Frequency**: 6 mentions
- **Key Themes**:
  - Urbanization
  - Deforestation
  - Land development
- **Numerical Value**: 50% of responses noted habitat loss

2. Predation and Invasive Species

Eleuthera:

- **Frequency**: 7 mentions
- **Key Themes**:
  - Raccoons

    - Wild animals eating crabs
- **Numerical Value**: 41% of responses mentioned predation

Andros:

- **Frequency**: 2 mentions
- **Key Themes**:
    - More predators
- **Numerical Value**: 17% of responses noted predation

New Providence:

- **Frequency**: 0 mentions
- **Numerical Value**: 0%

3. Harvesting Practices

Eleuthera:

- **Frequency**: 5 mentions
- **Key Themes**:
    - Over-harvesting
    - Crabbing out of season
    - Increased commercial demand
- **Numerical Value**: 29% of responses discussed harvesting issues

Andros:

- **Frequency**: 4 mentions
- **Key Themes**:
    - Catching spawning crabs
    - Taking reproducing females
- **Numerical Value**: 33% of responses mentioned harvesting

New Providence:

- **Frequency**: 1 mention
- **Key Themes**:
    - Limited commercial activity

- **Numerical Value**: 8% of responses noted harvesting

4. Population Perception

Eleuthera:

- **Frequency**: 4 mentions
- **Key Themes**:
  - More baby crabs
  - Crabs still present in deep bush
- **Numerical Value**: 24% of responses suggested stable/increasing population

Andros:

- **Frequency**: 2 mentions
- **Key Themes**:
  - No significant concern
  - Crabs still present
- **Numerical Value**: 17% of responses indicated stable population

New Providence:

- **Frequency**: 2 mentions
- **Key Themes**:
  - Faster reproduction
  - Still present but fewer
- **Numerical Value**: 17% of responses suggested mixed population status

Comparative Analysis

Primary Concerns by Island

1. **Eleuthera**: Habitat destruction and raccoon predation
2. **Andros**: Forest fires and disrupted reproductive cycles
3. **New Providence**: Urban development and habitat loss

Local Ecological Knowledge Insights

- **Cultural Practices**: Significant human intervention through harvesting
- **Traditional Harvesting**: Digging for crabs, seasonal harvesting

- **Ecological Observation**: Local fishers note specific environmental changes
- **Adaptation Strategies**: Suggestion that crabs can be found deeper in bushlands

Key Findings

- All three islands show signs of black crab population decline
- Habitat destruction is the most consistent theme across islands
- Local knowledge emphasizes complex ecological interactions
- Human activities (development, harvesting) significantly impact crab populations

Recommendations

1. Implement sustainable harvesting practices
2. Protect remaining habitat areas
3. Conduct more detailed population studies
4. Engage local communities in conservation efforts

Limitations: Survey relies on local perceptions and may lack scientific precision. Further scientific research is recommended to validate these findings.

Q 32, Where specifically are black land crabs found in the bush/forest?

Primary Habitat Categories

1. Cover/Concealment Elements (53.2%)

- Under rocks: 30.8% (28 responses)
- Under leaves/leaf litter: 13.2% (12 responses)
- Under logs/fallen trees: 6.6% (6 responses)
- Under generic "stuff"/structures: 2.2% (2 responses)
- Under porches: 1.1% (1 response)

2. Underground Features (37.4%)

- In holes/burrows: 18.7% (17 responses)
- In sinkholes/"banana holes": 9.9% (9 responses)
- In caves/crevasses: 3.3% (3 responses)
- Specific burrow characteristics: 5.5% (5 responses)

3. Vegetation Associations (36.3%)

- Near/under trees (general): 19.8% (18 responses)
- Specific tree species: 12.1% (11 responses)
- Near roots: 5.5% (5 responses)
- Among palm fronds: 2.2% (2 responses)

4. Forest/Bush Characteristics (29.7%)

- Deep within bush/forest: 15.4% (14 responses)
- Thick/dense forest/bush: 7.7% (7 responses)
- General "bush"/"forest"/"coppice": 6.6% (6 responses)

5. Moisture-Related Habitats (13.2%)

- Near water sources/ponds/lakes: 7.7% (7 responses)
- In marshy areas/swamps: 5.5% (5 responses)

6. Food Resource Associations (8.8%)

- Near fruit trees: 5.5% (5 responses)

- In gardens/near crops: 3.3% (3 responses)

7. Landscape Features (6.6%)

- Rocky areas (beyond hiding under individual rocks): 4.4% (4 responses)
- Leeward hillside: 1.1% (1 response)
- Coastal areas: 1.1% (1 response)

8. Specific Geographic Locations (3.3%)

- Bannerman Town area: 3.3% (3 responses)

9. Human-Modified Areas (3.3%)

- Abandoned homes: 1.1% (1 response)
- Cleared areas: 1.1% (1 response)
- Near roads: 2.2% (2 responses)

10. No Knowledge (3.3%)

- "Don't know"/"No idea": 3.3% (3 responses)

Local Ecological Knowledge Insights

1. Behavioral Observations

- **Movement Patterns**: "They tend to stay still when catching" indicates knowledge of defensive freeze behavior
- **Activity Levels**: "Move around the area more frequently" suggests observations of activity cycles
- **Camouflage Behavior**: "Use as camouflage" shows understanding of crab's adaptive strategies
- **Avoidance Behavior**: "Avoids swamps," "avoids ponds" indicates knowledge of habitat preferences

2. Microhabitat Selection

- **Temperature Preferences**: "Like areas that are nice and cool" suggests thermal regulation knowledge
- **Moisture Requirements**: "Moist places" indicates understanding of physiological needs
- **Shade Requirements**: "Shaded areas" suggests knowledge of desiccation avoidance
- **Seasonal Adaptations**: "You dig for crabs in the off season" indicates temporal knowledge

3. Burrow Characteristics

- **Depth Knowledge**: "Crab holes are like four feet deep" demonstrates specific measurement knowledge
- **Complex Underground Systems**: References to "limestone sinkholes, caves and crevasses" shows understanding of interconnected habitat use

4. Food Web Relationships

- **Diet Knowledge**: Association with specific fruit trees suggests understanding of crab feeding ecology
- **Garden Attraction**: "They like the veggies and fruit that grow" shows observation of feeding preferences

5. Species-Specific Knowledge

- **Black Crab Preferences**: Specific mention of "prevalence of black crabs around sinkholes" suggests species-specific habitat knowledge

Cultural Practice Insights

1. Traditional Ecological Knowledge

- **Tree Species Importance**: Specific mentions of "Fig Tree, Thatch Tree, Pigeon Plum Tree" and "bully mastic, poison-wood, pigeon plum" demonstrate detailed indigenous botanical knowledge
- **Local Terminology**: Use of terms like "banana holes" and "coppice" reflects regional terminology

2. Harvesting Strategies

- **Location-Based Tactics**: "Deep within the bush (Bannerman Town)" suggests area-specific knowledge
- **Opportunistic Harvesting**: "Just get them when they are out" indicates passive collection strategies
- **Deliberate Hunting**: "You dig for crabs in the off season" shows active pursuit strategies
- **Garden Trapping**: "Once you have a garden you can catch them easily" suggests using gardens as attraction points

3. Environmental Reading Skills

- **Habitat Assessment**: Recognition of "leeward hillside" as favorable habitat shows landscape-level ecological understanding
- **Development Impact Awareness**: "Remote less developed" areas preferred suggests understanding of human impact on crab populations

Particularly Notable Findings

1. **Sinkhole Importance**: The repeated mention of sinkholes (9.9%) suggests these are critical habitat features, particularly for black crabs, revealing specialized knowledge about karst topography utilization.
2. **Specific Tree Associations**: The detailed knowledge of tree species associations (12.1%) demonstrates sophisticated understanding of habitat relationships beyond simple cover requirements.
3. **Depth Perception**: References to "deep within the bush" (15.4%) suggests not just presence in forest but specific spatial understanding of where within forests crabs are more likely found.
4. **Temporal Knowledge**: Reference to "off season" digging indicates awareness of seasonal behavior patterns and adaptive harvesting techniques.
5. **Garden Relationships**: The observation that crabs are attracted to gardens suggests potential human-wildlife interactions and possibly intentional baiting techniques.
6. **Microhabitat Selection**: Understanding of crabs' preference for "cool" areas shows knowledge of thermal regulation behaviors.
7. **Geographic Specificity**: The mention of Bannerman Town specifically suggests possible local population concentrations or specialized harvesting areas.

Capture Techniques

- **Hunting Methods**:
  - "Dig for crabs"
  - "Can be easily found under a porch"
  - "Once you have a garden you can catch them easily"

Key Patterns and Synthesis

The analysis reveals that black land crabs in the Bahamas demonstrate clear habitat preferences that can be organized hierarchically:

1. **Primary Association**: Rocky areas (26.0%) and dense bush/forest (22.9%) represent the most frequently mentioned habitats
2. **Secondary Association**: Tree-associated habitats (21.9%) and holes/burrows (18.8%) form another significant habitat cluster
3. **Tertiary Association**: Leaf litter (14.6%) and fruiting trees (10.4%) create microhabitat preferences
4. **Local Knowledge Nuance**: The repeated emphasis on "depth" in habitat (deep in bush, deep holes) suggests crabs prefer secluded, protected environments away from disturbance

The data demonstrates sophisticated local ecological knowledge, particularly regarding:

- The relationship between crabs and specific tree species
- Precise geological features (distinguishing sinkholes from regular holes)
- Understanding of crab behavioral responses to environmental conditions (staying still, camouflage)
- Seasonal adaptations and foraging behaviors

This localized knowledge provides invaluable insights that complement scientific understanding of crab ecology and could inform conservation strategies that incorporate both traditional knowledge and western scientific approaches.

Q 38, Why is the bush/forest important for land crabs?

Primary Themes Identified

1. **Habitat/Home** (General living environment)
2. **Protection/Shelter** (Safety from predators, weather, humans)
3. **Food/Nutrition** (Feeding grounds, resource availability)
4. **Physical Characteristics** (Environmental conditions like shade, darkness, moisture)
5. **Reproduction** (Breeding, spawning)

Frequency Analysis

Theme 1: Habitat/Home (Generic References)

Total: 53 mentions (61.6%)

- "Home" variants: 20 mentions (23.3%)
- "Habitat" variants: 25 mentions (29.1%)
- "Where they live" variants: 8 mentions (9.3%)

Theme 2: Protection/Shelter

Total: 24 mentions (27.9%)

- Protection from predators: 10 mentions
- Protection from humans: 3 mentions
- Hiding places: 7 mentions
- Shelter (general): 4 mentions

Theme 3: Food/Nutrition

Total: 16 mentions (18.6%)

- General food references: 10 mentions
- Specific food items (berries, leaves, plants): 2 mentions
- Feeding grounds: 4 mentions

Theme 4: Physical Environment Characteristics

Total: 9 mentions (10.5%)

- Shade/coolness: 3 mentions
- Darkness/canopy: 2 mentions

- Ground suitable for burrowing: 2 mentions
- References to natural conditions: 2 mentions

Theme 5: Reproduction

Total: 2 mentions (2.3%)

- Breeding: 1 mention
- Spawning: 1 mention
-

Analysis of Response Patterns

1. **Simple vs. Complex Responses**:
   - Single-theme responses: 58 (67.4%)
   - Multi-theme responses: 28 (32.6%)
2. **Most Common Multi-Theme Combinations**:
   - Habitat + Food + Protection: 7 mentions
   - Habitat + Protection: 6 mentions
   - Habitat + Food: 5 mentions

Insights on Response Patterns

1. **Predominance of Basic Habitat References**: The majority of responses simply state that forests are the crabs' "home" or "habitat" without elaboration. This suggests a basic understanding that black land crabs are forest-dwelling species, but doesn't demonstrate deeper ecological knowledge.
2. **Protection Emphasis**: Many responses that go beyond basic habitat mention highlight protection from predators, suggesting respondents recognize the forest as a refuge. This indicates some understanding of predator-prey relationships and the protective function of forests.
3. **Food Source Recognition**: Nearly one-fifth of responses mention food availability, recognizing forests as resource-rich environments for crabs. Some responses specifically mention forest vegetation as food sources.
4. **Limited Understanding of Life Cycle**: Very few responses mention reproduction or life cycle aspects, suggesting limited awareness of the crabs' complete ecological needs beyond basic survival requirements.
5. **Environmental Conditions**: Some more detailed responses mention specific environmental conditions like shade, darkness, and suitable ground for burrowing, showing a more nuanced understanding of the crabs' specific habitat requirements.

Conclusion

The responses demonstrate varying levels of ecological understanding, with most being simple habitat statements. The more insightful responses recognize multiple forest functions: as shelter from predators and harsh conditions, as a food source, and as suitable terrain for burrowing. This analysis shows that while most respondents have a basic understanding that forests are the natural home for black land crabs, fewer demonstrate comprehensive knowledge of the specific ecological relationships and requirements these crabs have with their forest habitat.

Q 40, Is there anything else about land crabs that you would like to share with us?

1. Ecological Knowledge

Habitat & Behavior

- **Frequency**: 11 mentions
- **Percentage**: 19.6% of total responses
- **Key Themes**:
  - "Crabs like the dry areas"
  - "Crabs don't like vibration, sound or heat"
  - "The backs of crabs are always wet (moisture)"
  - "Crabs march twice a year"
  - "They molt underground in their burrows"
  - "Baby crabs live near to adults in holes, juveniles are everywhere"

Species Interactions

- **Frequency**: 9 mentions
- **Percentage**: 16.1% of total responses
- **Key Themes**:
  - Predator-prey relationships: "Herons eat the crabs" (2 mentions)
  - Interspecies competition: "Black and White crabs hate each other" (2 mentions)
  - "White crabs hate black crabs and kill them" (2 mentions)
  - "White crabs are more dominant"
  - "Not keeping black crabs and white crabs in the same bucket/pen"

Environmental Connections

- **Frequency**: 6 mentions
- **Percentage**: 10.7% of total responses
- **Key Themes**:
  - "High tide is important to release the eggs"
  - "The decline in crabs in Northern Eleuthera is due to invasive raccoons" (2 mentions)

    - "Taking the bush away to build houses and hotels might change the way they live"
    - "Some farmers will use the crab feces for fertilizer"

2. Cultural & Culinary Knowledge

Culinary Practices

- **Frequency**: 7 mentions
- **Percentage**: 12.5% of total responses
- **Key Themes**:
    - "All types of different recipes, minced, crab'n rice, etc."
    - "Crab'n Rice is causing too many people to crab, its just too good"
    - "They taste good and there is a lot of different dishes you can cook"
    - "Different cultures in the Bahamas cook the crabs differently"
    - "Crabs are different in size and taste from island to island"
    - "They taste really good"

Cultural Significance

- **Frequency**: 8 mentions
- **Percentage**: 14.3% of total responses
- **Key Themes**:
    - "Our culture, generation to generation"
    - "Houses built off crabbing in Bannerman town"
    - "Bannerman town is the mecca of crabs"
    - "Andros is the crab capital and crab fest"
    - "It is important for the youth to learn"
    - "Crabbing is great exercise and fun" (2 mentions)

3. Harvesting & Management Practices

Harvesting Techniques

- **Frequency**: 8 mentions
- **Percentage**: 14.3% of total responses

- **Key Themes**:
  - "Digging for crabs harms them off season"
  - "You dig for crab during the off season"
  - "Groups are good for tactically catching crabs, like herding them"
  - "Crabs can be easily found under a porch"
  - "Catch crabs from behind so they don't pinch"
  - "If the crabs bite me I bite them back"
  - "Once you have a garden you can catch them easily"
  - "Crabs can be caught in one's personal garden, they like the veggies and fruit that grow"

Conservation & Management

- **Frequency**: 7 mentions
- **Percentage**: 12.5% of total responses
- **Key Themes**:
  - "The crabs should be protected during the molting season"
  - "Crabs are good for Bahamians but should be controlled as to not be a pest for farming"
  - "Rules of selling crab based on the property, not the government"
  - "Concerns about private property and restricting access to crabbing"
  - "Lot of resentment towards the Haitian community of using 'Bahamian Resources'"
  - "Not as many people are crabbing, many are working. The need for jobs is greater"
  - "I think crab is less important in younger generation. Most of the crabbers are older generation"

Local Ecological Knowledge Analysis

Traditional Ecological Knowledge Systems

1. **Phenological Knowledge**:
   - Understanding of crab life cycles: "Crabs march twice a year"

- Recognition of molting periods and reproduction cycles
- Knowledge of tidal influence: "High tide is important to release the eggs"

2. **Species-Specific Knowledge**:
   - Detailed understanding of interspecies dynamics between black and white crabs
   - Recognition of habitat preferences: "Crabs like the dry areas"
   - Behavioral observations: "White crabs are faster, they never stop running once seen"
3. **Spatial Knowledge**:
   - Geographic specificity: "Bannerman town is the mecca of crabs"
   - "Used to be crabs up North Eleuthera before the racoons ate them"
   - "Andros is the crab capital"
   - Island-specific differences: "Crabs are different in size and taste from island to island"
4. **Observer Knowledge**:
   - Anatomical observations: "The backs of crabs are always wet (moisture)"
   - Defensive behaviors: "Not the big pincher but the small one that hurts more"
   - "The smaller biter hurts more than the larger biter"

Cultural Knowledge Integration

1. **Intergenerational Knowledge Transfer**:
   - "Our culture, generation to generation"
   - "It is important for the youth to learn"
   - Concern about generational shifts: "crab is less important in younger generation"
2. **Economic Significance**:
   - "Houses built off crabbing in Bannerman town"
   - Competition for resources: "Lot of resentment towards the Haitian community"
   - Changing economic priorities: "The need for jobs is greater"
3. **Resource Management Tensions**:
   - Official vs. unofficial management: "Rules of selling crab based on the property, not the government"

- Conservation awareness: "The crabs should be protected during the molting season"
- Access concerns: "Concerns about private property and restricting access to crabbing"

Key Insights

1. **Complex Socio-ecological System**: The data reveals a sophisticated understanding of crab ecology embedded within cultural practices and economic considerations. Bahamians demonstrate detailed knowledge of multiple crab species, their behaviors, and their ecological roles.
2. **Environmental Change Awareness**: Respondents show awareness of anthropogenic impacts on crab populations, noting habitat destruction and invasive species as significant threats.
3. **Cultural Identity**: Land crabs represent more than just a food source—they are intertwined with cultural identity, economic livelihoods, and traditional knowledge systems.
4. **Intergenerational Knowledge Concerns**: There is clear anxiety about the transmission of traditional crabbing knowledge to younger generations as economic priorities shift.
5. **Resource Management Conflicts**: Tensions exist around access to crabbing grounds, management authority, and resource competition between different communities.
6. **Holistic Understanding**: The knowledge system demonstrates remarkable integration of biological, ecological, cultural, and economic dimensions, revealing a holistic understanding of land crabs within Bahamian society.
